# Aging at the Crossroads of Cuproptosis and Ferroptosis: From Molecular Pathways to Age-Related Pathologies and Therapeutic Perspectives

**DOI:** 10.3390/ijms27010522

**Published:** 2026-01-04

**Authors:** Grażyna Gromadzka, Beata Tarnacka, Magdalena Cieślik

**Affiliations:** 1 Department of Biomedical Sciences, Faculty of Medicine, Collegium Medicum, Cardinal Stefan Wyszynski University in Warsaw, Wóycickiego Street 1/3, 01-938 Warsaw, Poland; 2 Department of Rehabilitation, Eleonora Reicher National Institute of Geriatrics, Rheumatology and Rehabilitation, Spartańska 1, 02-637 Warsaw, Poland; 3 Department of Rehabilitation Medicine, Faculty of Medicine, Warsaw Medical University, Spartańska 1, 02-637 Warsaw, Poland; 4 Department of Cellular Signalling, Mossakowski Medical Research Institute, Polish Academy of Sciences, Pawińskiego 5, 02-106 Warsaw, Poland

**Keywords:** aging, Alzheimer’s disease, cuproptosis, cardiovascular disease, cancer, curcumin, diet, ferroptosis, hormesis, infection, inflammaging, neurodegeneration, oxidative stress, Parkinson’s disease, polyphenols, polyunsaturated fatty acids (PUFAs)

## Abstract

Aging is a multifactorial process marked by a progressive decline in physiological function and increased vulnerability to diseases such as neurodegeneration, cancer, cardiovascular disorders, and infections. A central feature of aging is inflammaging, a state of chronic low-grade inflammation driven by cellular senescence, mitochondrial dysfunction, and oxidative stress. Recently, two regulated forms of non-apoptotic cell death—ferroptosis and cuproptosis—have emerged as critical mechanisms linking redox imbalance, mitochondrial stress, and disrupted metal homeostasis to age-related pathology. Ferroptosis, an iron-dependent process characterized by lipid peroxidation and impaired glutathione peroxidase 4 (GPX4) activity, and cuproptosis, a copper-dependent mechanism associated with protein lipoylation stress, both intersect with aging-related changes in mitochondrial and metabolic function. Importantly, these two forms of cell death should not be viewed as entirely separate pathways but rather as interconnected axes within a broader metal–redox–metabolic network. Disturbances in copper or iron homeostasis, glutathione (GSH)/GPX4 dysfunction, mitochondrial and iron-sulfur (Fe–S) cluster compromise, and enhanced lipid peroxidation may converge to lower cellular survival thresholds, thereby exacerbating oxidative damage, immune dysfunction, and tissue degeneration and ultimately fueling aging and inflammaging. This review offers a unique integrated perspective that situates ferroptosis and cuproptosis within a unified framework of aging biology, emphasizing their roles in age-related diseases and the therapeutic potential of targeting these pathways through nutritional, pharmacological, and lifestyle interventions.

## 1. Introduction

Aging is a complex and multifactorial biological process characterized by a progressive decline in physiological functions and increased susceptibility to a wide range of diseases, including neurodegeneration, cancer, cardiovascular disorders, and infections [1,2]. One of the hallmarks of aging is the chronic, low-grade systemic inflammation that develops in the absence of overt infection—a phenomenon termed inflammaging [3,4]. Although the exact triggers of inflammaging are still being elucidated, cellular senescence, mitochondrial dysfunction, and oxidative stress are considered major contributors [5].

In recent years, two distinct and regulated forms of non-apoptotic cell death—ferroptosis and cuproptosis—have emerged as critical mechanisms involved in redox imbalance and mitochondrial stress [6,7,8]. Ferroptosis, first described in 2012, is an iron-dependent form of cell death driven by excessive lipid peroxidation and depletion of glutathione peroxidase 4 (GPX4) activity [9]. On the other hand, cuproptosis, reported in 2022, is a novel copper-dependent form of cell death that is triggered by the accumulation of intracellular copper ions and dysregulated mitochondrial protein lipoylation, leading to proteotoxic stress [10].

Both ferroptosis and cuproptosis are intimately connected to metabolic and mitochondrial pathways that are dysregulated with aging [11,12]. Age-related changes in iron and copper homeostasis have been associated with increased oxidative damage, inflammation/neuroinflammation, and impaired cellular viability [13,14,15]. Furthermore, these forms of regulated cell death may actively contribute to the maintenance of chronic inflammation in older individuals by releasing damage-associated molecular patterns (DAMPs), exacerbating immune dysfunction and tissue degeneration [8].

This review explores the emerging roles of ferroptosis and cuproptosis in the biology of aging. We discuss how disturbances in iron and copper metabolism contribute to these pathways and examine their implications in age-related diseases. Finally, we highlight the potential of nutritional and pharmacological strategies targeting ferroptosis and cuproptosis as novel interventions to mitigate aging-associated pathologies.

While previous reviews have typically addressed ferroptosis and cuproptosis separately or within the context of specific diseases, the present work provides an integrated perspective that unites these two metal-dependent regulated cell death pathways within a single framework of aging biology. By comparing their molecular mechanisms, shared redox and metabolic features, and implications for inflammaging, this review aims to bridge the current knowledge gap and highlight the intertwined roles of iron- and copper-induced cell death in age-related degeneration. This integrative approach distinguishes our work from earlier reviews and underscores its contribution to understanding how redox-active metals shape the aging process.

## 2. Review Methodology

This narrative review was conducted to synthesize current knowledge on the roles of ferroptosis and cuproptosis in aging and inflammaging, with a focus on their molecular mechanisms, involvement in age-related diseases, and therapeutic potential. A comprehensive literature search was performed in PubMed, Scopus, and Web of Science databases from inception to October 2025. The following keywords and their combinations were used: “ferroptosis”, “cuproptosis”, “regulated cell death”, “aging”, “inflammaging”, “iron metabolism”, “copper metabolism”, “oxidative stress”, “mitochondrial dysfunction”, and “age-related diseases” (e.g., “Alzheimer’s disease”, “cardiovascular disease”, “cancer”, “macular degeneration”, “neurodegeneration”).

Both original research articles and review papers published in English were considered. Priority was given to peer-reviewed studies that provided mechanistic insights into the links between regulated cell death and aging processes. Preprints and non-peer-reviewed sources were excluded. Reference lists of selected articles were manually screened to identify additional relevant publications.

Given the emerging nature of cuproptosis, particular attention was paid to experimental studies and recent reviews published after 2022. Data were critically analyzed and organized into thematic sections that reflect the current state of knowledge, controversies, and gaps in understanding. The findings are presented in a structured manner, highlighting both shared and distinct features of ferroptosis and cuproptosis in the context of aging and chronic inflammation.

## 3. Ferroptosis and Cuproptosis: Molecular Mechanisms

Ferroptosis and cuproptosis are two recently characterized forms of regulated cell death (RCD), each dependent on the intracellular accumulation of specific redox-active metals—iron and copper, respectively. Although distinct in their triggers and execution, both share critical features such as metabolic dependency, oxidative stress, and mitochondrial dysfunction, which make them particularly relevant to aging and age-related diseases.

### 3.1. Key Molecular Pathways

Ferroptosis, an iron- and lipotoxicity-dependent RCD, was initially observed in 2003 using erastin to selectively kill RAS (rat sarcoma virus)-mutant cancer cells [16]. Stockwell group formally defined it in 2012 as iron-dependent, non-apoptotic RCD [9,17]. Mechanisms that trigger and inhibit ferroptosis are related to the availability of iron or its carriers and the proper functioning of lipophilic antioxidants. Ferroptosis is mediated by reactive oxygen species (ROS) and is characterized by the concomitant accumulation of intracellular iron and the occurrence of lipid peroxidation. This process is mechanistically driven by iron-catalysed lipid peroxidation, mediated through Fenton reactions and lipoxygenases (LOXs), targeting polyunsaturated fatty acids (PUFAs) possessing two or more double bonds within their aliphatic chain [18]. It has been demonstrated that administration of erastin, a small-molecule ferroptosis inducer, inhibits cystine uptake, leading to glutathione (GSH) depletion and subsequent inactivation of the phospholipid repair enzyme GPX4 [19]. GPX4 catalyses the reduction of potentially cytotoxic lipid hydroperoxides (L-OOH) into non-deleterious lipid alcohols (L-OH) [20]. Ablation of GPX4 activity culminates in robust lipid peroxidation and subsequent cellular death. Therefore, the mitigation of lipid peroxidation is essential for mammalian cells to preclude ferroptosis, a process that can initiate intrinsically upon GPX4 inactivation. GPX4 normally uses GSH to reduce membrane lipid hydroperoxides, but its loss leads to an accumulation of peroxidized phospholipids [21]. The structural consequences include membrane thinning and increased curvature, as supported by molecular dynamics simulations. The structural consequences include membrane thinning and increased curvature, as supported by molecular dynamics simulations. These perturbations weaken lipid packing and can trigger a positive feedback loop: as curvature and disorder increase, the membrane becomes more susceptible to further oxidation, eventually leading to pore formation [22].

Molecular-dynamics simulations and model membrane experiments support the following mechanistic chain: oxidation of PUFA-containing phospholipids (e.g., via GPX4 inactivation) leads to accumulation of lipid hydroperoxide or aldehyde moieties in the sn-2 acyl chains (fatty acid chains attached to the second carbon atom of a glycerol backbone), which reorient toward the aqueous interface and form hydrogen bonds with headgroups or water. This conformational shift increases the average area per lipid while reducing bilayer thickness and tail ordering—thereby thinning the membrane [23]. Simultaneously, such oxidised lipids alter the spontaneous curvature of the leaflet: in giant unilamellar vesicles subject to asymmetric oxidation, the targeted leaflet undergoes shape transitions (budding, prolate–to-oblate) consistent with a decrease in preferred area and increased curvature [24]

These two effects—thinning and increased curvature—then establish a positive-feedback loop: membrane thinning and curvature increase expose deeper acyl-chains and enhance access by oxidants, promoting further lipid peroxidation; further peroxidation exacerbates thinning/curvature, weakening bilayer mechanical integrity, reducing breakthrough force and line-tension, and eventually facilitating pore formation or micellization [18,25]. Therefore, lipophilic radical scavengers (e.g., vitamin E, Fer-1, Lip-1) may interrupt this cycle by trapping lipid radicals, stabilising bilayer architecture and preventing the cascade of thinning-curvature–oxidation-destabilisation [26,27,28]. Furthermore, cytotoxic aldehydes, such as 4-hydroxynonenal (4-HNE) and malondialdehyde (MDA), generated as byproducts of lipid hydroperoxides, may contribute to cellular demise through the formation of protein crosslinks [9,18,29]. Moreover, ferroptosis exhibit distinctive mitochondrial morphological alterations, characterized by dysmorphic mitochondria with reduced or absent cristae and ruptured outer mitochondrial membranes. These ultrastructural changes, observed through transmission electron microscopy, are a hallmark of ferroptosis and distinguish it from other forms of regulated cell death [30]. The involvement of pro-apoptotic B-cell lymphoma 2 (BCL2) family members, specifically Bcl-2 homology 3 (BH3)-interacting domain death agonist (BID) and BCL2-binding component 3 (BBC3, also known as p53 upregulated modulator of apoptosis (PUMA)), in modulating these mitochondrial changes has been suggested, indicating a potential interplay between apoptotic and ferroptotic pathways. However, ferroptosis proceeds independently of the canonical apoptosis executioners, BCL-2-associated X (BAX) and BCL-2-antagonist killer (BAK), further emphasizing its unique mechanistic underpinnings [9]. Specifically, the loss of mitochondrial cristae indicates disruption of oxidative phosphorylation and energy production, while the rupture of the outer mitochondrial membrane may facilitate the release of pro-oxidant molecules, such as iron, into the cytosol, thus propagating lipid peroxidation [30,31]. What is more, the BID and PUMA, implicates these BH3-only proteins in sensitizing cells to ferroptosis, possibly by altering mitochondrial membrane permeability [32]. However, the precise mechanisms by which these proteins contribute to mitochondrial dysfunction in ferroptosis remain an area of active investigation. The independence of ferroptosis from BAX and BAK, as demonstrated by Dixon et al. [9], clarifies that the mitochondrial perturbations are not solely a consequence of canonical apoptosis-mediated mitochondrial outer membrane permeabilization.

A study by Tsvetkov and colleagues showed that intracellular copper induces a novel form of RCD, different from oxidative stress-related cell death such as apoptosis, ferroptosis, or necroptosis, and has been termed “cuproptosis” [8]. Cuproptosis is initiated by the direct binding of copper to lipoylated components of the tricarboxylic acid (TCA) cycle, which causes aggregation of lipoylated mitochondrial proteins, loss of iron–sulfur cluster proteins and proteotoxic stress, ultimately leading to mitochondrial dysfunction and cell death. Although mitochondrial impairment during cuproptosis can secondarily increase reactive oxygen species (ROS) production, oxidative stress is a downstream consequence rather than the initiating event of cuproptosis [33].

The hallmark of cuproptosis is the direct binding of copper ions to lipoylated components of the TCA cycle, known as the Krebs cycle, notably dihydrolipoyl acetyltransferase (DLAT), a key enzyme within the pyruvate dehydrogenase complex (PDC). This interaction triggers the aggregation of these lipoylated proteins, disrupting their enzymatic activity and impairing mitochondrial respiration [8]. Lipoylation, a crucial post-translational modification, is essential for the function of several mitochondrial enzymes involved in energy production. Specifically, the lipoyl moiety acts as a cofactor in the PDC and α-ketoglutarate dehydrogenase (KGD), facilitating acyl group transfer. Excessive copper binding to these lipoylated components disrupts this process, leading to the formation of toxic protein aggregates. This aggregation not only impedes the normal flow of metabolites through the TCA cycle but also induces mitochondrial stress, ultimately compromising adenosine triphosphate (ATP) production [8,10]. Furthermore, cuproptosis involves the disruption of iron–sulfur (Fe-S) cluster biogenesis. Fe-S clusters are essential cofactors for a wide array of mitochondrial proteins involved in electron transport, oxidative phosphorylation, and various metabolic pathways. Copper overload can interfere with the assembly and stability of these clusters, further exacerbating mitochondrial dysfunction. The combined effects of lipoylated protein aggregation and Fe-S cluster disruption create a synergistic toxic environment within the mitochondria, leading to severe cellular damage. The resulting mitochondrial dysfunction manifests as impaired respiration, increased ROS production, and ultimately, cell death. This copper-mediated cell death pathway highlights the critical role of copper homeostasis in cellular survival [31].

### 3.2. Crosstalk Between Ferroptosis and Cuproptosis Pathways

Recent evidence strongly supports a bidirectional coupling between copper homeostasis and ferroptosis sensitivity. On one side, excess copper promotes ferroptosis: for example, copper directly binds to GPX4 at Cys107 and Cys148, leading to its ubiquitination, aggregate formation and macroautophagic degradation via the receptor Tax1-binding protein 1 (TAX1BP1)—thereby lowering the threshold for ferroptotic lipid peroxidation [34].

Conversely, copper deficiency also sensitises cells to ferroptosis: depletion of copper using the chelator bathocuproine disulfonate (BCS) causes mitochondrial perturbation, GSH depletion, lowered GPX4 protein expression and enhanced susceptibility to ferroptotic triggers [35]. Mechanistically, the intersection of these pathways can be outlined as follows: copper and iron metabolism converge on mitochondrial and redox circuits. In the case of cuproptosis, copper overload binds lipoylated enzymes of the TCA-cycle (e.g., DLAT), causes loss of Fe–S cluster proteins, disrupts electron transport, elevates ROS and perturbs iron handling [36,37].

In the ferroptotic axis, iron-dependent lipid peroxidation, GSH/GPX4 axis collapse, and redox collapse are central. The fact that copper modulates GPX4 stability or GSH availability places it upstream of the ferroptotic cascade. Further, oxidative damage to cuproproteins (for example, oxidation of ceruloplasmin (CP)) may liberate copper and thereby feed into cuproptotic or ferroptotic triggers (iron mis-handling and ROS amplification)—indicating a plausible feed-forward loop: lipid peroxidation-cuproprotein oxidation-copper release-mitochondrial stress/Fe–S protein loss–enhanced lipid peroxidation [30,37,38].

From the perspective of ageing and age-related disease, this crosstalk has special relevance: mitochondrial dysfunction, redox imbalance, accumulation of labile copper and iron pools, and decreased capacity of antioxidant systems (GSH, GPX4, nuclear factor erythroid 2-related factor 2 (NRF2)-axis) are hallmarks of cellular senescence and tissue ageing. As such, a perturbation of copper homeostasis may lower the ferroptotic threshold and/or tip cells into cuproptotic stress in aged tissues, contributing to pathologies such as neurodegeneration, cardiovascular disease or osteoarthritis. Indeed, in chondrocytes from osteoarthritic cartilage copper excess acts via down-regulation of solute carrier family 7 member 11 (SLC7A11), ROS-mediated lipid peroxidation and increases ferroptosis susceptibility [39].

Therefore, the intersection of cuproptosis and ferroptosis should be considered not as two totally separate death pathways, but as interconnected axes within the broader metal–redox–metabolic network. Disturbances in copper or iron homeostasis, GSH/GPX4 dysfunction, mitochondrial/Fe–S cluster compromise and lipid peroxidation can all converge to lower cell survival thresholds in ageing or disease states.

## 4. Aging

**Figure 1:**
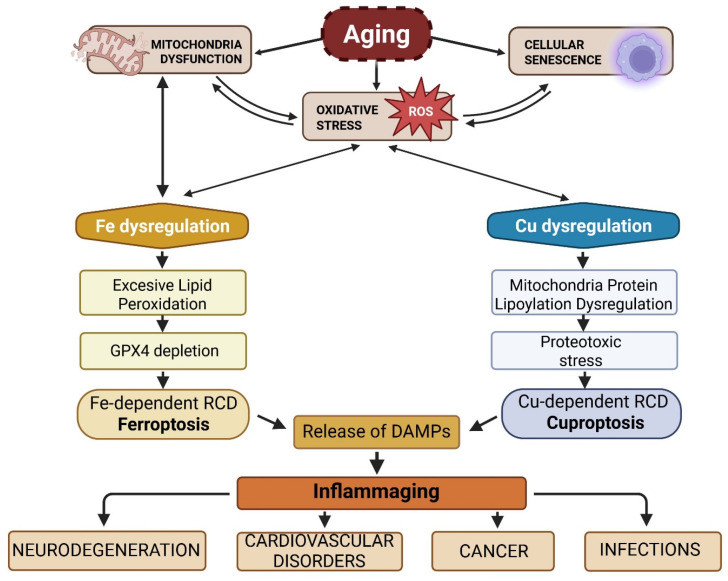
***Aging-driven mitochondrial dysfunction and cellular senescence elevate oxidative stress (ROS), disrupting iron and copper homeostasis.*** Fe dysregulation enhances lipid peroxidation and depletes GPX4, culminating in ferroptosis. Cu dysregulation impairs mitochondrial protein lipoylation and induces proteotoxic stress, triggering cuproptosis. Both regulated cell death pathways release DAMPs that amplify inflammaging, thereby promoting neurodegeneration, cardiovascular disorders, cancer, and infection susceptibility. Abbreviations: DAMPs, danger-associated molecular patterns; GPX4, glutathione peroxidase 4; RDC, regulated cell death; ROS, reactive oxygen species. Figure created with BioRender.com.

Aging, a pleiotropic process characterized by the progressive decrement of physiological function, augmented oxidative stress, chronic inflammatory states, and heightened disease susceptibility [40] is marked by the accrual of cellular damage and the disruption of cellular homeostasis. This process exhibits interindividual variability modulated by a multitude of endogenous and exogenous factors [41]. While the precise mechanisms driving aging remain incompletely understood, recent investigations have implicated cuproptosis and ferroptosis, as potential contributors. The exact roles of these metal-dependent RCD pathways in aging are still under scrutiny, It is hypothesized that the time-dependent accumulation of metals, including iron or copper, may exacerbate age-related cellular damage and tissue dysfunction [42,43] (Figure 1).

These pathways underscore the importance of maintaining cellular homeostasis and mitigating cellular damage accrual as crucial strategies for promoting healthy aging [44]. Furthermore, these RCD pathways are intricately linked to “inflammaging,” a chronic, low-grade inflammatory state that develops with age [3,45,46,47].

### 4.1. Mitochondrial and Oxidative Stress in Aging

According to the mitochondrial free radical theory of aging, inefficiency and damage in mitochondrial electron transport chain (ETC) complexes—particularly complexes I and III—lead to increased electron leakage and the generation of ROS such as superoxide anion (O2^•−^), hydrogen peroxide (H_2_O_2_), and hydroxyl radicals (^•^OH) [1,48,49]. These ROS arise when electrons prematurely reduce molecular oxygen, a process exacerbated by age-related declines in ETC efficiency and the accumulation of mitochondrial DNA (mtDNA) mutations. Oxidative lesions in mtDNA impair the synthesis of ETC subunits encoded by the mitochondrial genome, further compromising electron transfer and ATP production [50,51]. The origin of mtDNA mutations is multifactorial: firstly, mtDNA is located in close proximity to the inner membrane where ROS are generated, and exhibits limited DNA repair capacity, making it vulnerable to oxidative base lesions and strand breaks [52]. Secondly, errors during mtDNA replication—driven by the mitochondrial DNA polymerase γ and influenced by mitochondrial nucleoids and mitophagy dynamics—contribute significantly to the mutation burden, especially under conditions of impaired mitochondrial turnover [53].

As mtDNA mutations accumulate, they result in defective ETC-encoded subunits, reduced oxidative phosphorylation efficiency and increased electron leak, thereby feeding back to further ROS production and mitochondrial damage [51,54]. The reduced activity of antioxidant defences such as manganese superoxide dismutase (MnSOD), GPXs, and peroxiredoxins aggravates ROS accumulation, and in fact age-associated studies demonstrate lowered enzymatic activities or protein levels of these systems in older subjects—for example, erythrocyte GPx activity declines with advancing age [55], and mitochondrial peroxiredoxin 6 (Prx6) levels are reduced in aged tissues [56]. Such declines impair ROS detoxification, reduce protection against oxidative damage and thus contribute to elevated oxidative burden in aging cells and tissues [57].

As mitochondrial electron-transport-chain (ETC) performance deteriorates—particularly via reduced activity or damage in complexes I/III—electron leakage increases and generates reactive oxygen species (ROS) such as O_2_•^−^, H_2_O_2_ and •OH [54]. This ROS overload causes oxidative damage to lipids, proteins and DNA, and undermines mitochondrial bioenergetics, leading to ATP deficit, redox imbalance and mitochondrial dysfunction. In turn, mitochondrial dysfunction exacerbates ROS generation, creating a self-amplifying cycle of oxidative stress and bioenergetic failure [58]. Crucially, in this context ferroptosis may be triggered because elevated mitochondrial ROS and impaired antioxidant defences (e.g., diminished GSH/GPX4 activity) facilitate iron- and lipid-mediated peroxidation of membrane phospholipids—a hallmark of ferroptosis [59]. Thus, collapse of ETC activity drives excessive mitochondrial ROS formation, destabilizes Fe–S cluster biogenesis (for example via reduced NFS1 activity), and increases the redox-active mitochondrial iron pool, collectively disrupting lipid/iron redox equilibrium and facilitating peroxidation of PUFA-containing phospholipids—the obligate substrates of ferroptosis. These events trigger canonical ferroptotic signaling by amplifying Fenton chemistry and lipid radical propagation, providing a molecularly defined axis that links mitochondrial decline to ferroptotic pathway activation and enhanced cellular susceptibility (e.g., NFS1 depletion sensitizes cells to ferroptosis via iron accumulation and oxidative stress [60]; mitochondrial ROS and labile iron drive ferroptosis through Fenton reactions [61].

Antioxidant defences, including glutathione peroxidase, superoxide dismutase, and peroxiredoxins, decline with age, reducing the cell’s ability to detoxify ROS and repair oxidative damage [48,62,63]. Impairment of mitochondrial antioxidant defences, especially reductions in peroxiredoxin 6 within metabolically active tissues with high mitochondrial requirements, has been directly linked to increased H_2_O_2_ formation and lipid peroxidation during aging [62].

### 4.2. Link Between Mitochondrial and Oxidative Stress, and Ferro-/Cuproptosis

The progressive loss of metal homeostasis during aging has profound implications for redox balance and cell fate. Iron and copper are redox-active transition metals that readily cycle between reduced and oxidized states (Fe^2+^/Fe^3+^ and Cu^+^/Cu^2+^), enabling them to participate in essential enzymatic reactions but also rendering them potent catalysts of ROS formation through Fenton and Haber–Weiss chemistry. Under conditions of dyshomeostasis, excessive labile iron or copper pools drive uncontrolled lipid peroxidation and protein oxidation, thereby sensitizing cells to RCD [64]. The mechanisms underlying this metal-driven form of cell death, including the detailed pathways of ferroptosis and cuproptosis, will be discussed in depth in the following sections.

In ferroptosis, excess intracellular iron contributes to the generation of hydroxyl radicals via the Fenton reaction and fuels iron-dependent lipoxygenases, resulting in peroxidation of polyunsaturated phospholipids. Impaired antioxidant defences, particularly depletion of GSH and inactivation of GPX4, exacerbate this process, establishing a lethal lipid ROS burden [65].

As it was mentioned above, cuproptosis arises when copper overload disrupts mitochondrial metabolism by binding to lipoylated TCA-cycle proteins, triggering their aggregation, loss of Fe–S enzymes, and consequent proteotoxic stress distinct from the lipid-peroxidation-driven nature of ferroptosis [8,10]. With aging, systemic copper accumulation and diminished copper clearance capacity may potentiate this pathway, particularly in metabolically active tissues with high mitochondrial demand, such as the heart, kidney, and brain, which rely heavily on mitochondrial respiration and are known to accumulate copper in pathological or age-related contexts. In addition, age-related mitochondrial decline may alter the lipoylation status of TCA cycle enzymes and compromise Fe–S cluster stability, thereby amplifying the susceptibility of aged cells to copper-induced stress [66]. In neurodegenerative disorders, where disrupted copper homeostasis is a well-established feature, these mechanisms are increasingly recognized as contributing factors to cuproptosis-associated neuronal loss [67].

With aging, cumulative oxidative damage to mitochondria, decline in antioxidant capacity, and disrupted metal trafficking systems synergize to amplify susceptibility to ferroptosis and cuproptosis. Thus, iron and copper dyshomeostasis not only accelerate oxidative stress but also serve as pivotal triggers of these regulated cell death pathways, linking transition metal biology to tissue degeneration in aging [42,68].

### 4.3. Chronic Inflammation in Aging

Cellular senescence is a state in which cells undergo a stable cell-cycle arrest and no longer proliferate despite remaining metabolically active. This growth arrest is enforced by the upregulation of cyclin-dependent kinase inhibitors—most notably p16^INK4a (CDKN2A) and p21^CIP1/WAF1 (CDKN1A)—which suppress cyclin-dependent kinase (CDK) activity, activate the retinoblastoma (RB) pathway, and block cell-cycle progression through G1 (and, in some contexts, G2) into S phase. In contrast to quiescent cells, senescent cells do not readily re-enter the cell cycle in response to mitogenic signals. This cell-cycle exit is accompanied by profound changes in chromatin organization, persistent DNA damage responses and metabolic rewiring. Senescent cells also develop a senescence-associated secretory phenotype (SASP), a complex secretome including pro-inflammatory cytokines (e.g., interleukin-1 beta (IL-1β), IL-6, IL-8), chemokines, growth factors and proteases—that can act in a paracrine manner to promote local inflammation, extracellular matrix remodelling and immune cell recruitment. Importantly, experimental studies in aged animals and human tissues show that senescent cells accumulate with chronological age and contribute causally to tissue dysfunction: selective removal of p16^INK4a-expressing cells improves multiple age-related phenotypes in mice, and pharmacological senolytic interventions that reduce senescent cell burden ameliorate frailty and other functional deficits. Together, persistent senescent cell accumulation and SASP production provide a mechanistic basis connecting cellular growth arrest with organismal inflammaging and age-related functional deterioration [69,70,71,72,73].

Age-related mitochondrial dysfunction enhances the release of DAMPs, amplifying a vicious cycle of cellular injury and immune activation. DAMPs—endogenous molecules released from stressed or damaged cells, including mtDNA, ATP, high-mobility group box 1 (HMGB1), and oxidized lipids—activate pattern recognition receptors (PRRs) such as Toll-like receptors (TLRs), the receptor for advanced glycation end-products (RAGE), and the cyclic GMP–AMP synthase–stimulator of interferon genes (cGAS–STING) pathway, as well as the NOD-, LRR- and pyrin domain-containing protein 3 (NLRP3) inflammasome. Activation of these pathways promotes the production of pro-inflammatory cytokines, notably IL-1β and IL-18, thereby sustaining chronic inflammation. As mitochondrial dysfunction progresses with age, increased DAMP release further intensifies PRR signaling, perpetuating the cycle of inflammatory amplification [74,75,76,77].

The NLRP3 inflammasome is a critical cytosolic sensor of the innate immune system, activated by a wide range of pathogen-associated molecular patterns (PAMPs) and DAMPs. Its activation typically requires a two-step process: a priming signal, most often mediated through nuclear factor kappa-light-chain-enhancer of activated B cells (NF-κB) activation downstream of TLRs or cytokine receptors, which induces transcription of NLRP3 and pro–IL-1β, followed by an activation signal provided by diverse cellular perturbations such as potassium efflux, lysosomal destabilization, or mitochondrial dysfunction [78,79].

Following its assembly, the NLRP3 inflammasome recruits and activates caspase-1, leading to the processing of pro–IL-1β and pro–IL-18 into their active cytokines and the induction of gasdermin D–dependent pyroptosis, collectively amplifying inflammatory responses [80]. During aging, cumulative mitochondrial damage may promote excessive ROS production and the release of oxidized mitochondrial DNA into the cytosol, both of which serve as potent triggers of NLRP3 activation [81,82]. Concurrently, the age-dependent accumulation of DAMPs (e.g., extracellular ATP, uric acid crystals, advanced glycation end-products) acts on pattern-recognition receptors, while aged myeloid cells themselves exhibit a heightened basal activation state due not only to cumulative mitochondrial ROS production, impaired mitophagy, altered NAD^+^ metabolism, and chromatin remodelling that enforces a “primed” NF-κB transcriptional landscape, but also to defective autophagy, reduced clearance of damaged cellular components, and chronic exposure to systemic pro-inflammatory cues such as SASP factors from senescent cells. These processes collectively maintain inflammatory genes in a pre-activated configuration, diminish the resolution of inflammation, and ultimately lower the activation threshold for NLRP3 inflammasome assembly [83,84]. This age-related enhancement of NLRP3 activity sustains a chronic, low-grade proinflammatory milieu-inflammaging, characterized by persistent elevations in circulating cytokines such as tumour necrosis factor-α (TNF-α), IL-6, and IL-1β, which collectively contribute to tissue dysfunction and the pathogenesis of multiple age-associated diseases [85,86].

### 4.4. Ferroptosis and Cuproptosis as Missing Links in Inflammaging

Inflammaging emerges from the interplay of senescent cell burden, DAMP-induced innate immune activation, and immune aging. Regulated cell death pathways—ferroptosis and cuproptosis—act at this intersection. Age associated metal dyshomeostasis, oxidative stress, and mitochondrial dysfunction predispose cells to metal-dependent death, which may further amplify inflammation via DAMP release and inflammasome activation. Whether these death pathways ultimately promote or restrain inflammaging depends on context: the spatial distribution of dying cells, immune competence, and the efficiency of cell clearance mechanisms [87,88,89,90].

As mentioned above, ferroptosis, marked by lipid peroxidation and GPX4 suppression, can both fuel and mitigate inflammaging. On one hand, ferroptosis releases DAMPs such as oxidized phospholipids and mitochondrial fragments, which can activate PRRs and trigger NLRP3 inflammasome assembly, thereby amplifying chronic inflammation [91,92]. Furthermore, in aging tissues where iron accumulates and antioxidant capacity (e.g., GPX4, GSH) declines, cells are more prone to ferroptotic death, exacerbating tissue dysfunction and inflammatory cytokine release [93]. Conversely, in certain contexts, ferroptosis may eliminate damaged or senescent cells that contribute to SASP production, potentially reducing local inflammation if clearance is efficient and immune responses intact. The net effect likely depends on the balance between ferroptotic clearance and secondary inflammatory activation [94].

Moreover, cuproptosis induces mitochondrial proteotoxic stress and DAMP-mediated NLRP3 activation while disrupting iron metabolism, thereby increasing susceptibility to ferroptosis [8,10]. Notably, aging is associated with copper dyshomeostasis—both excess and impaired mitochondrial copper handling—which creates conditions conducive to cuproptosis in metabolically active cells [43]. The relative influence of ferroptosis versus cuproptosis in inflammaging remains under active investigation: emerging studies indicate crosstalk between the two pathways, e.g., ferroptosis inducers (FINs), increasing copper sensitivity via GSH depletion and enhanced lipoylation [66].

Collectively, ferroptosis and cuproptosis act as mechanistic amplifiers of inflammaging by generating immunogenic signals that reinforce SASP cytokine networks, enhance mitochondrial ROS production, and disrupt iron and copper trafficking, thereby lowering the threshold for further ferroptotic and cuproptotic initiation [95,96]. This bidirectional crosstalk RDC and inflammaging establishes a self-perpetuating pathogenic loop that drives tissue degeneration and loss of homeostatic capacity during aging. Importantly, ferroptotic and cuproptotic cells not only undergo regulated demise but also function as upstream propagators of inflammation through the release of oxidized lipids, DAMPs, and mitochondrial constituents [97,98,99]. Thus, these forms of RCD should be viewed not as terminal events but as dynamic processes that fuel chronic inflammatory signaling and accelerate age-related pathology [65,100,101].

## 5. Ferroptosis and Cuproptosis in Aging: Disease Associations

Age-related diseases are characterized by increased oxidative stress, mitochondrial dysfunction, and chronic low-grade inflammation—all of which converge on dysregulated forms of cell death such as ferroptosis and cuproptosis. These mechanisms are increasingly recognized as key drivers in the pathogenesis of neurodegeneration, cardiovascular diseases, cancer, and severe infections like coronavirus disease 2019 (COVID-19), especially in older adults.

### 5.1. Neurodegenerative Diseases

Ferroptosis has emerged as a central mechanism of neuronal death in several neurodegenerative disorders, including Alzheimer’s disease (AD), Parkinson’s disease (PD), Huntington’s disease (HD), and others. Neurons are particularly susceptible to ferroptosis due to their unique metabolic profile: high oxygen consumption, abundant mitochondrial activity, elevated intracellular iron levels, and the presence of PUFAs in neuronal membranes that are prone to lipid peroxidation [102,103,104].

In AD, iron accumulation and increased lipid peroxidation products have been consistently observed in the hippocampus and cerebral cortex, correlating with cognitive impairment [105,106]. Neuropathological and experimental studies demonstrate that iron dyshomeostasis is a central pathogenic feature of AD, particularly in the hippocampus and cortex, where impaired iron export—such as reduced ferroportin expression—and increased ferritin collectively expand the labile ferrous iron pool and potentiate Fenton chemistry–driven formation of ROS [107,108,109,110]. This excess iron directly accelerates lipid peroxidation, leading to the elevated production of 4-HNE and MDA, which damage neuronal membranes and proteins and are consistently found at higher concentrations in AD brain regions vulnerable to degeneration [107,111,112]. Compounding this biochemical vulnerability, AD brains demonstrate profound depletion of critical antioxidant systems, including GSH and GPX4, with pronounced deficits observed within lipid rafts of the hippocampus and cortex. This reduction substantially compromises neuronal detoxification of lipid hydroperoxides, thereby heightening regional susceptibility to iron-mediated oxidative injury [111,113,114,115].

The convergence of iron overload, increased lipid peroxidation, and impaired antioxidant defenses aligns with growing evidence that ferroptosis, an iron-dependent, lipid-peroxide-driven form of RDC, contributes directly to neuronal loss and progressive cognitive impairment in AD [113,115]. Human post-mortem studies further substantiate these mechanisms by revealing significantly elevated iron levels in hippocampal and cortical tissues, with iron burden strongly correlating with accelerated cognitive decline [110,114]. Biomarker analyses show parallel increases in 4-HNE and MDA in these regions, which are associated with memory deficits and disease severity [109,112,113], while neuroimaging modalities such as MRI and quantitative susceptibility mapping confirm regional iron accumulation that predicts cognitive performance [108,110,114]. Complementary evidence from AD mouse models demonstrates iron buildup and heightened lipid peroxidation in cortex and hippocampus, and interventions that reduce iron content or inhibit ferroptosis—such as iron chelators, GPX4 overexpression, or activation of the Xc^−^/GPX4 pathway—consistently mitigate neurodegeneration and improve cognitive outcomes [113,115,116].

Collectively, these findings support a mechanistic model in which iron accumulation, oxidative lipid damage, and compromised antioxidant defenses interact to drive ferroptosis and, ultimately, the cognitive deterioration characteristic of AD [109,116,117].

Similarly, in PD, dopaminergic neurons of the substantia nigra pars compacta (SNpc), exhibit pathological iron deposition and reduced antioxidant defenses, contributing to oxidative injury. Neuroimaging and post-mortem pathological studies consistently demonstrate substantial iron accumulation within the SNpc—particularly in vulnerable dopaminergic neurons—strongly implicating iron dysregulation as a key driver of PD pathogenesis and progression [118,119,120].

A large body of evidence indicates that iron metabolism is profoundly disturbed in PD, with multiple mechanisms—including increased uptake, impaired storage, and defective export—potentially driving pathological iron loading in vulnerable dopaminergic neurons, thereby enhancing intracellular iron availability and promoting iron-dependent ROS formation [121]. Although iron is indispensable for neuronal and glial cell function, excessive labile iron facilitates oxidative damage, and alterations in iron concentrations within the substantia nigra are thought to play a key role in the selective degeneration of dopaminergic neurons in PD [122]. These neurons exhibit heightened susceptibility to oxidative injury due to age-related declines in antioxidant defenses, continuous exposure to ROS, and the presence of endogenous neurotoxic metabolites such as ortho-quinones; moreover, iron and ROS amplify one another’s accumulation, establishing a self-propagating oxidative stress cycle [123].

Clinical studies consistently reveal elevated oxidative stress markers (e.g., thiobarbituric acid reactive substances (TBARS), advanced oxidation protein products (AOPP)) and reduced antioxidant capacity—including lower ferric reducing antioxidant power (FRAP) values, vitamin C concentration, and non-protein thiol content—in individuals with PD, further supporting the role of iron-driven redox imbalance in PD pathology [124].

Accumulating evidence from both clinical and experimental studies implicates dysregulated copper homeostasis in the pathogenesis of AD and PD. Human meta-analyses and biomarker studies report altered systemic copper in AD (often increased serum/plasma Cu) and region-specific decreases in copper in postmortem brains from AD and PD dementia cases, indicating disease- and compartment-specific copper dyshomeostasis [125]. Mechanistic and biophysical studies show that copper interacts directly with disease-relevant proteins: copper promotes amyloid-β redox activity and aggregation in AD models and binds to α-synuclein, modulating its conformation and aggregation propensity in PD-relevant systems [126,127,128]. Experimental models further support pathogenic roles for copper: perturbations of copper handling (genetic or environmental) exacerbate neurodegeneration in cellular and organismal systems, and copper dyshomeostasis is linked to mitochondrial dysfunction, oxidative stress, and lipid peroxidation—mechanisms that can precipitate neuronal loss [129]. Thus, integrative investigations combining metallomic speciation with functional assays in longitudinal human cohorts and well-characterized models are needed to determine whether modulating copper homeostasis represents a viable therapeutic strategy in AD and PD. The specific role of copper in these neurodegenerative conditions is discussed in detail in a separate manuscript prepared by our group [67].

Growing evidence indicates that ferroptosis plays a significant pathogenic role in HD, where mutant huntingtin (mHTT) enhances oxidative stress and disrupts neuronal iron homeostasis, rendering striatal projection neurons particularly susceptible to this iron-dependent, lipid-peroxidation–driven form of cell death [130,131,132]. Hallmark biochemical changes—including GSH depletion, GPX4 inactivation, and accumulation of lipid peroxides—are consistently observed in HD mouse models (e.g., R6/2, N171) and in human post-mortem tissue, accompanied by ferroptosis-related transcriptional alterations revealed by single-nucleus RNA sequencing of the HD striatum [133,134].

Mechanistic studies demonstrate that ferroptotic stress in HD is exacerbated by impaired mitochondrial bioenergetics, increased labile iron originating from dysfunctional iron–sulfur cluster biogenesis, and dysregulation of lipid-metabolizing enzymes such as acyl-CoA synthetase long-chain family member 4 (ACSL4) and arachidonate 5-lipoxygenase (ALOX5), which promote peroxidation of PUFA-containing phospholipids. Importantly, pharmacological inhibition of ferroptosis provides functional neuroprotection in HD models. Fer-1 reduces striatal neuron death in HD organotypic slices and protects against mHTT-induced oxidative lipid damage [135]. More recent work shows that ALOX5-driven lipid peroxidation contributes directly to HD neurotoxicity and that Fer-1 rescues the viability of HD neural cells by suppressing ALOX5-mediated ferroptosis [135,136].

In parallel, cuproptosis—a copper-dependent, iron-independent form of RCD driven by copper interactions with lipoylated mitochondrial proteins—has been increasingly implicated in HD as well as in additional pathological contexts, including PD and AD. Human and animal studies demonstrate elevated striatal copper levels in HD and show that copper exacerbates mHTT aggregation and toxicity. Excess copper promotes mitochondrial proteotoxic stress by destabilizing lipoylated components of the TCA cycle and impairing oxidative phosphorylation. In vivo, copper enhances mHTT aggregation and accelerates neurodegeneration in HD models [137]. Copper chelation mitigates these defects: ammonium tetrathiomolybdate delays onset of motor symptoms and reduces neurotoxicity in R6/2 mice [134], and abnormal striatal copper accumulation was identified in HD human tissue and mouse models [138]. These findings demonstrate that copper dyshomeostasis and cuproptotic stress interact with mitochondrial dysfunction and oxidative injury to worsen HD pathology.

Microglial dysfunction is a critical driver of neurodegenerative disease progression, as impaired immune surveillance, disrupted metal homeostasis, and maladaptive inflammatory signaling synergistically accelerate neuronal vulnerability and loss. Importantly, microglia are now recognized as active participants in ferroptotic pathways rather than passive responders. Microglia possess one of the highest iron storage capacities among brain cell types, leading to their unusually high susceptibility to ferroptosis and significant iron accumulation in neurodegenerative diseases [139,140]. This heightened sensitivity is evidenced by primary murine microglia undergoing iron-dependent lipid peroxidation and death at significantly lower concentrations of inducers RSL3, erastin, buthionine sulfoximine (BSO) compared with astrocytes, an effect entirely preventable by inhibitors such as Lip-1 or deferiprone [141]. Their vulnerability is further amplified by stress-induced iron accumulation and transcriptional reprogramming, including protein transport protein Sec24 homolog B (SEC24B)-dependent regulation of ferroptotic sensitivity [142].

Ferroptotic microglia release pro-inflammatory cytokines and DAMPs, fueling sustained neuroinflammation and creating a permissive environment for neuronal degeneration, a phenomenon especially detrimental during aging and inflammaging [143]. Additionally, microglial polarization alters ferroptotic sensitivity: M2 microglia are more prone to ferroptosis than M1 microglia, likely due to differences in ferritin buffering and labile iron content [144]. Importantly, both ferroptosis and cuproptosis can release mitochondrial and lipid-derived DAMPs that further activate microglia and astrocytes, establishing a self-amplifying inflammatory loop [145].

Given that dysregulation of both iron and copper metabolism constitutes a shared pathogenic hallmark across neurodegenerative diseases, the pronounced sensitivity of microglia to these metal-dependent death pathways creates a mechanistic nexus linking redox imbalance, metal dyshomeostasis, innate immune activation, and progressive neuronal degeneration [141].

### 5.2. Cardiovascular Diseases

Aging is a major risk factor for cardiovascular diseases (CVD), and regulated forms of cell death—particularly ferroptosis and cuproptosis—are increasingly recognized as key contributors to cardiac and vascular pathology. Ferroptosis plays a central role in cardiomyocyte death, endothelial dysfunction, and atherogenesis. In murine models of doxorubicin-induced cardiomyopathy and ischemia/reperfusion (I/R) injury, ferroptosis has been demonstrated as a significant mode of regulated cell death: for example, treatment with the ferroptosis inhibitor Fer-1 or iron chelation markedly reduced cardiomyocyte death, preserved mitochondrial integrity, and improved cardiac function [146,147].

In a myocardial infarction (MI) model induced by left anterior descending (LAD) coronary artery ligation in mice, administration of Fer-1 lowered levels of redox-active (ferrous) iron, reduced lipid peroxidation (MDA), restored GSH, and enhanced expression of GPX4 and cystine/glutamate antiporter (a transporter that imports cystine into the cell in exchange for glutamate) xCT encoded by the *SLC7A11* gene, thereby attenuating infarct size and myocardial injury [148].

Moreover, genetic studies have identified key endogenous regulators that determine how susceptible the aging heart is to ferroptosis. One example is uncoupling protein-2 (UCP2), a mitochondrial inner-membrane protein involved in controlling reactive oxygen species production and maintaining mitochondrial redox balance. In mice lacking UCP2, I/R injury triggers significantly more severe ferroptosis, characterized by greater accumulation of redox-active iron, increased expression of ACSL4—a lipid-metabolic enzyme that promotes incorporation of polyunsaturated fatty acids into membrane phospholipids, thereby making them susceptible to peroxidation—and reduced levels of GPX4, the central antioxidant enzyme that detoxifies lipid hydroperoxides. These changes translate into markedly elevated indices of lipid peroxidation and worse post-ischemic cardiac function. Importantly, pharmacological inhibition of ferroptosis with Fer-1 partially rescued cardiac injury in UCP2-deficient mice, demonstrating that UCP2 normally acts as an intrinsic brake on ferroptotic damage during cardiac stress [149]. Moreover, non-coding RNA regulation has also been implicated in modulating ferroptotic susceptibility in the heart. A circular RNA named FEACR (ferroptosis-associated circRNA) is highly relevant in this context: in cardiomyocytes, overexpression of FEACR reduces iron-mediated lipid peroxidation and cell death during hypoxia/reoxygenation (H/R), and in vivo, it reduces infarct size after ischemia–reperfusion (I/R) injury.

Mechanistically, FEACR physically interacts with nicotinamide phosphoribosyltransferase (NAMPT), stabilizing its protein level, which leads to increased activity of sirtuin 1 (SIRT1) (a NAD^+^-dependent deacetylase). SIRT1 then deacetylates and activates forkhead box O1 (FOXO1), a transcription factor that induces expression of ferritin heavy chain 1 (FTH1), a key iron-storage protein. Upregulation of FTH1 limits the pool of redox-active iron, thereby suppressing ferroptosis in cardiomyocytes. This FEACR/NAMPT/SIRT1/FOXO1/FTH1 axis represents a promising regulatory circuit and potential therapeutic target for ferroptosis-linked cardiac injury [150].

In addition to ferroptosis, cuproptosis also contributes to cardiovascular aging. Although copper is essential for mitochondrial enzymes and redox balance, its dysregulation can become deleterious: in a mouse model of doxorubicin-induced cardiomyopathy, overexpression of metallothionein (MT)—a heavy metal scavenger—significantly mitigated cardiac remodeling, mitochondrial injury, and cell death. The authors observed that doxorubicin elevated expression of the copper importer: copper transporter 1 (CTR1), depressed the copper exporter: ATPase copper transporting beta (ATP7B), and altered levels of lipoylated mitochondrial proteins (such as DLAT), consistent with the activation of cuproptosis. Importantly, treatment with a copper chelator, tetrathiomolybdate, recapitulated the protective effect of MT; conversely, a copper ionophore (elesclomol) negated MT’s benefit [151].

Epidemiologically, elevated circulating copper levels, which are frequently reported in older individuals, may synergize with mitochondrial vulnerability in aged cardiomyocytes to trigger or exacerbate cuproptotic death—contributing to conditions such as heart failure with preserved ejection fraction (HFpEF), hypertrophic cardiomyopathy, or diabetic cardiomyopathy [152]. These pathological processes are further amplified by inflammaging—the chronic, low-grade activation of inflammatory pathways in aging. For instance, activation of the NLRP3 inflammasome in aging or stressed cardiac cells can enhance oxidative stress and drive ferroptotic or cuproptotic damage, thereby creating a vicious cycle of cell death, inflammation, and tissue remodeling [153].

Therapeutically, a multifaceted approach is warranted. Pharmacological inhibition of ferroptosis (e.g., Fer-1), copper chelation, and antioxidant therapies constitute promising strategies. For example, in a murine model of HFpEF induced by high-fat diet plus N(G)-Nitro-L-arginine methyl ester (L-NAME), both Fer-1 and the iron chelator deferiprone (DFP) lowered myocardial iron content, reduced ROS, increased superoxide dismutase (SOD) activity, preserved mitochondrial ultrastructure, and restored GPX4, xCT, and NRF2 expression [154].

Dietary modulation—limiting red meat and excessive iron or copper supplementation while favoring antioxidant-rich, polyphenol-dense foods—may help mitigate ferroptotic and cuproptotic stress by reducing systemic exposure to redox-active metals and enhancing dietary antioxidant capacity [155].

In parallel, lifestyle interventions such as regular aerobic exercise and modest caloric restriction exert well-documented benefits on mitochondrial health in the aging cardiovascular system. Both interventions activate the AMP-activated protein kinase (AMPK)–SIRT1–peroxisome proliferator-activated receptor-gamma coactivator 1-alpha (PGC-1α) axis, a central metabolic and stress–response pathway that coordinates mitochondrial biogenesis, promotes mitochondrial dynamics and mitophagy, and enhances cellular redox homeostasis. AMPK sensing of energetic stress leads to SIRT1 activation, which in turn deacetylates and activates PGC-1α—the master regulator of mitochondrial quality control. Through this pathway, exercise and caloric restriction also augment endogenous antioxidant defenses, including Nrf2-driven transcriptional responses, thereby lowering oxidative damage and improving vascular and myocardial resilience in aging organisms [156,157,158,159,160,161].

### 5.3. Cancer

Aging reshapes metal homeostasis, redox balance and mitochondrial function in ways that are highly relevant to tumor biology. Ferroptosis and cuproptosis intersect with cancer initiation, progression and therapy response, and their relevance may change with age [10,16,21]. Below we summarize current evidence linking these pathways to cancer in the context of aging, with precise citations to primary and recent review literature.

As previously demonstrated, ferroptosis is defined by iron-dependent accumulation of membrane lipid hydroperoxides when cellular antioxidant defenses (notably the cystine–GSH–GPX4 axis and parallel systems such as ferroptosis suppressor protein 1 (FSP1)/coenzyme Q10 (CoQ) and dihydroorotate dehydrogenase (quinone) (DHODH) are overwhelmed [16,21]. Many cancer types—in particular mesenchymal/dedifferentiated, therapy-resistant subpopulations—display metabolic and iron-handling phenotypes that make them especially susceptible to FINs, and preclinical data indicate that induction of ferroptosis can reverse resistance to chemotherapy, targeted agents and some immunotherapies [162,163,164]. Importantly, the role of ferroptosis in cancer is context-dependent: while ferroptotic death of malignant cells can be tumor-suppressive, ferroptosis of stromal or immune cells or ferroptosis-driven necroinflammation may paradoxically promote tumor progression in certain settings [165,166].

As it was mentioned above, cuproptosis is triggered by intramitochondrial copper binding to lipoylated components of the TCA cycle, provoking lipoylated-protein aggregation, loss of Fe–S cluster proteins, proteotoxic stress and cell death. Tumor cells with high mitochondrial respiration/dependency on lipoylated TCA enzymes appear particularly vulnerable [167]. Since the initial mechanistic description, several groups have explored copper-modulating agents (e.g., copper ionophores, copper-delivering nanomedicines) as anticancer strategies and profiled tumors for cuproptosis-sensitivity signatures. Since the initial mechanistic description, several groups have explored copper-modulating agents (e.g., copper ionophores, copper-delivering nanomedicines) as anticancer strategies and profiled tumors for cuproptosis-sensitivity signatures [168,169,170,171,172,173].

These signatures typically comprise cuproptosis-related genes (CRGs)—for example, *FDX1*, *DLAT*, lipoyltransferase 1 (*LIPT1*), glycine cleavage system H-protein (*GCSH*), solute carrier family 31 (copper transporter), member 1 (*SLC31A1*), and *ATP7B*—whose expression levels, copy-number variations, DNA methylation status or mutation burden are collected in large cohorts. Unsupervised clustering (e.g., consensus clustering) or supervised modelling (e.g., LASSO-Cox regression) is used to stratify tumors into “high-signature” vs. “low-signature” groups. For instance, one study used expression of 15 CRGs to define two clusters in ovarian cancer (C1 vs. C2) and a derived 8-gene risk score that correlated with overall survival [174].

The measurement pipeline is as follows: (1) select CRGs based on mechanistic knowledge of cuproptosis; (2) extract their mRNA expression (or methylation/copy number variation (CNV)/mutation) from bulk-tumor datasets; (3) apply statistical selection (univariate Cox, least absolute shrinkage and selection operator (LASSO)) to derive a minimal gene set; (4) compute a risk score per patient as a weighted sum of the gene expression levels; (5) validate the score in independent cohorts with Kaplan–Meier survival, compute area under the receiver operating characteristic curve (ROC-AUC) and sometimes treatment-response correlation. For example, in lung adenocarcinoma a cuproptosis-score predicted survival, immune checkpoint expression and drug sensitivity [170,171] [173,175].

Tumors exhibiting a high cuproptosis signature—for example, characterized by high expression of the copper importer SLC31A1, low levels of the copper exporter ATP7B, and elevated expression of lipoylation enzymes—may represent a metabolic vulnerability to copper-induced cell death and, therefore, could be more responsive to therapeutic strategies that increase intracellular copper, such as copper ionophores or copper-delivering nanomedicines. In addition, this cuproptosis signature often associates with features of the tumor immune microenvironment, including tumor mutational burden (TMB) and immune cell infiltration, which can influence both the tumor’s sensitivity to immunotherapies and its overall susceptibility to metal-dependent cytotoxic strategies. Hence, integrating cuproptosis signatures with immune and pharmacogenomic profiling may help identify patient subgroups most likely to benefit from copper-targeted interventions [172,176,177,178,179].

In a pan-cancer analysis across 20 tumor types, a 13-gene CRG signature was associated with mutation burden and poor survival in several cancers [173].

In summary, the cuproptosis-sensitivity signature concept provides a translational bridge: mechanistic insight—biomarker stratification—therapeutic targeting. Its measurement requires transcriptomic (and sometimes epigenetic) profiling of CRGs, risk-score calculation, and validation of survival/treatment response associations. Incorporation of such signatures into copper-modulating therapeutic trials may help identify the patients most likely to benefit.

#### 5.3.1. Aging–Metal Biology and Cancer Vulnerability

Multiple lines of evidence indicate that systemic and tissue iron handling changes with age [180,181,182].

Aging is associated with progressive tissue iron accumulation and disturbed ferritinophagy/iron-recycling, leading to an expanded labile iron pool (LIP) and greater propensity for Fenton chemistry and lipid peroxidation—both central drivers of ferroptosis. The LIP is defined as the pool of redox-active (non-protein-bound) Fe^2+^ and Fe^3+^ within cells that is readily available to participate in redox reactions (for instance, via Fenton reaction: Fe^2+^ + H_2_O_2_ → Fe^3+^ + •OH + OH^−^). Age-related declines in autophagy and lysosomal degradation impair the process of ferritinophagy (nuclear receptor coactivator 4 (NCOA4)-mediated ferritin turnover), causing ferritin to sequester iron but fail to release it, thereby increasing cellular iron burden and LIP [109,183].

Meanwhile, senescent cells show altered expression of iron regulatory proteins—increased transferrin receptor 1 (TfR1) and reduced ferroportin (FPN1) localization at the plasma membrane—which enhances iron uptake and reduces efflux, respectively [181,183]. The result is elevated intracellular redox-active iron that promotes Fenton chemistry, generating •OH, which in turn initiate lipid peroxidation of PUFA-containing phospholipids, thereby lowering the threshold for ferroptotic death [184].

Age-dependent alterations in copper metabolism have also been described; for example, the multicenter study found that serum CP exhibited oxidative modifications and reduced ferroxidase activity in older individuals, which can impair copper export and iron–copper interplay [185]. Changes in copper chaperones (such as antioxidant 1 copper chaperone (ATOX1)) and transporters (e.g., CTR1, ATP7B) are evident in aged tissues, leading to disrupted intracellular copper-trafficking and altered mitochondrial copper delivery [186]. Perturbations in copper availability or trafficking can influence mitochondrial function (by affecting cytochrome c oxidase and SOD1 maturation) and impair antioxidant systems (particularly the GPX4/GSH axis) that modulate ferroptotic sensitivity [187].

Notably, experimental copper depletion via the chelator Bathocuproine disulfonate (BCS) has been shown to reduce cellular GSH levels and lower GPX4 protein expression, thereby increasing ferroptotic vulnerability in vitro [35]. Thus, in aged tissues the combination of expanded LIP, enhanced lipid peroxidation, compromised GPX4/GSH antioxidant defences, and altered copper homeostasis creates a permissive environment for ferroptosis activation, which may modify both tumor cell and stromal susceptibility as well as the tumor microenvironment.

#### 5.3.2. Crosstalk Between the Two Pathways and Implications for Cancer in Older Individuals

Mechanistic and review literature increasingly points to cross-regulation between iron- and copper-dependent networks. Cuproptosis involves loss of Fe–S cluster proteins and proteotoxic mitochondrial stress, which may secondarily perturb cellular iron distribution and ROS generation [10]. Conversely, copper status modulates antioxidant capacity: experimental copper depletion lowered GPX4 protein and GSH and potentiated ferroptosis in cell models, implying that copper deficiency (or dysregulated copper trafficking) could sensitize cells to ferroptotic death [36]. A broad synthesis of Fe/Cu biology highlights multiple molecular interfaces—redox chemistry, shared cofactors, impacts on mitochondrial metabolism and proteostasis—through which the two pathways may potentiate each other under stress conditions (including those encountered in aging tissues) [66].

In the context of cancer, these interactions matter for two reasons. First, aged tissues and aged hosts commonly exhibit altered iron and copper homeostasis (increased tissue iron, dysregulated copper handling and mitochondrial dysfunction), which can change the baseline oxidative and metabolic state of both transformed cells and the tumor microenvironment and thus alter the efficacy and toxicity profile of ferroptosis- or cuproptosis-directed therapies [8]. Second, tumor cell heterogeneity means that some cancer cell clones (e.g., those with high mitochondrial oxidative phosphorylation (OXPHOS) and lipoylated TCA activity) may be preferentially susceptible to cuproptosis, whereas others (iron-addicted, mesenchymal clones) are more vulnerable to ferroptosis; age-related systemic changes might therefore skew which cell populations respond to metal-centric treatments [10,164,188].

#### 5.3.3. Translational Outlook and Cautions

Therapeutic exploitation of ferroptosis and cuproptosis in cancer—for example, combining FINs or copper ionophores with conventional therapies or using targeted delivery systems—is an active area of preclinical and early translational work [189]. However, in older patients the altered systemic iron/copper milieu and increased burden of senescent cells may change the therapeutic window: increased tissue iron could enhance tumor killing but also raise risks of collateral tissue ferroptosis and necroinflammation, while copper modulation could impair antioxidant defenses (GPX4/GSH) in non-tumor tissues [34,104]. Therefore, rational biomarker-driven selection (tumor metabolic/cuproptosis or ferroptosis signatures, host iron/copper status) and careful evaluation of age-specific safety will be essential as these modalities move toward the clinic [104,189,190].

### 5.4. COVID-19 and Other Infections

Elderly individuals are particularly vulnerable to infections such as COVID-19, influenza and sepsis, experiencing higher morbidity and mortality due to impaired immune responses (immunosenescence), dysregulated inflammation (inflammaging) and reduced antioxidant capacity. Studies have demonstrated that older adults have diminished naïve T-cell pools, attenuated B-cell responses, impaired neutrophil and macrophage function, and slower vaccine-induced antibody production [191,192]. Coupled with chronic low-grade inflammation and oxidative stress, this immune decline creates a pro-oxidative environment that exacerbates tissue damage during acute infections [193]. For example, in COVID-19 older patients (>70 yrs) showed markedly higher case-fatality rates, prolonged hospital stays, and severe outcomes compared to younger cohorts, even when adjusting for comorbidities [194]. Similarly, seasonal influenza leads to disproportionately high hospitalization and death rates among persons aged ≥65, underscoring the effect of age-related immune weakening [195].

Thus, age-related immunosenescence, inflammaging, and mitochondrial dysfunction create a pro-oxidative environment that amplifies tissue damage during acute infections [196,197]. Recent evidence has identified ferroptosis as a key contributor to SARS-CoV-2-induced pathology [196,198,199]. Severe COVID-19 is characterized by a cytokine-driven acute-phase response in which IL-6–mediated induction of hepcidin redistributes iron into storage compartments, producing hypoferremia with concurrent hyperferritinemia; multiple clinical cohorts report that elevated hepcidin and ferritin strongly correlate with disease severity and mortality [200,201]. Local perturbations of iron handling are also evident in affected tissues: autopsy lung analyses from fatal COVID-19 cases demonstrate increased ferritin light chain (FTL), upregulation of transferrin receptor 1 (TFRC/TfR1), and elevated lipid-peroxidation products (MDA, 4-HNE), findings consistent with local iron accumulation and ferroptotic injury [202]. Single-cell and bulk transcriptomic profiling of severe COVID-19 lungs further reveal upregulation of iron-regulatory transcripts (FTL, FTH1, TFRC) in pulmonary epithelial cells and macrophages, supporting a picture of tissue-specific iron dysregulation and enhanced ferroptotic vulnerability [203,204]. Mechanistic experiments corroborate these observations. In vitro, lung epithelial cells exposed to inflammatory stimuli or iron loading (ferric/ammonium citrate) develop increased lipid peroxidation, downregulation of ferroptosis-protective proteins (GPX4, SLC7A11), and features of ferroptotic death; these effects are attenuated by ferroptosis inhibitors such as Fer-1 and Lip-1 [205,206]. Likewise, human endothelial cells treated with serum from COVID-19 non-survivors show increased ROS, elevated MDA/4-HNE, decreased GPX4, SLC7A11 and FTH1, all of which are reversible by Fer-1 or by blocking TNFR1 signalling [207]. Finally, clinical biomarker studies identify subsets of critically ill COVID-19 patients with signatures of iron dyshomeostasis and systemic lipid peroxidation that correlate with organ injury and worse outcomes [208]. These findings support a model in which SARS-CoV-2–associated systemic inflammation together with hepcidin-driven iron sequestration expands labile iron pools that catalyse Fenton chemistry and lipid peroxidation; when antioxidant defences (notably GPX4/GSH) are compromised, this biochemical milieu favours ferroptotic cell death in vulnerable pulmonary and vascular compartments. Pharmacological ferroptosis inhibitors protect against organ injury in several preclinical models of SARS-CoV-2 or related lung-injury models, indicating translational potential [203,209,210].

In parallel, dysregulation of copper metabolism may also contribute to COVID-19 pathogenesis and severity. CP, the major copper-binding ferroxidase in plasma that con-verts Fe(II) to Fe(III) and facilitates iron export, behaves as an acute-phase reactant and is frequently altered during systemic inflammation and viral infection; clinical series in COVID-19 report disturbed circulating copper/CP levels that track with disease severity [211]. Mechanistically, copper has a dual, context-dependent role in host–pathogen biology. At physiological levels copper is required for multiple antimicrobial and immune functions (e.g., cuproenzymes in phagocytes, support of T-cell responses), and copper surfaces/exposure have demonstrable antiviral activity against coronaviruses in vitro and on fomites [212,213].

Conversely, pathologic intracellular copper overload triggers a recently described, mitochondria-dependent form of regulated cell death—cuproptosis—in which copper binds directly to lipoylated mitochondrial enzymes, causing their aggregation, loss of iron–sulfur cluster proteins, proteotoxic stress and cell death. Genetic and chemical perturbation experiments (ferredoxin 1 (FDX1), lipoic acid synthetase (LIAS), lipoylation machinery, and copper ionophores such as elesclomol) establish that excess copper can therefore be cytotoxic by this distinct mechanism [8,162]. Thus, both extremes of copper imbalance are potentially harmful in infection: copper deficiency impairs innate and adaptive immune responses (reduced neutrophil function, impaired T-cell proliferation and lower production of key cytokines), increasing susceptibility to infection and weakening host defence, whereas copper excess can precipitate mitochondrial proteotoxicity and cell death that may worsen tissue injury [214,215].

Age-associated changes in copper handling—including altered CP dynamics and impaired hepatic copper export—could therefore amplify COVID-19 severity by simultaneously weakening antiviral immunity and increasing vulnerability of metabolically active cells to copper-driven injury. Integrating measurements of circulating copper and CP with tissue/cellular markers of cuproptosis (e.g., FDX1, lipoylation signatures) may help stratify patients in whom copper-modulating interventions (either supplementation or chelation/ionophore strategies) would be beneficial or harmful. Beyond COVID-19, a growing literature links ferroptosis to sepsis and infection-related organ failure. Experimental models (lipopolysaccharide (LPS), cecal ligation and puncture (CLP) and pathogen-specific models) reveal GPX4 downregulation, increased lipid peroxidation and iron redistribution in affected organs; pharmacologic or genetic inhibition of ferroptosis (e.g., Fer-1, liproxstatin-1, GPX4 preservation) reduces tissue damage and improves outcomes in multiple preclinical sepsis models [216]. Mechanistic studies demonstrate that during infection or inflammatory stress, ferritinophagy—selective autophagic degradation of ferritin mediated by the cargo receptor NCOA4—can be pathologically activated. Ferritinophagy releases stored iron from ferritin into the labile iron pool, elevating intracellular redox-active iron that fuels lethal lipid peroxidation in both parenchymal and immune cells [198,217]. In macrophages, this process is particularly critical: tissue-resident or M2-like macrophages displaying high ferritinophagy activity (via NCOA4) show increased labile iron and are more susceptible to ferroptosis, as shown in knockout or knockdown models (Atg5 or NCOA4). When ferritinophagy is blocked (e.g., via NCOA4 deletion), labile iron levels fall, lipid peroxidation is reduced, and ferroptotic cell death is prevented [198].

During pathogen-driven inflammation, such elevated labile iron and ferroptotic stress may impair immune effector cells, reducing their survival and functionality, thereby contributing to immune dysregulation, particularly in aged hosts whose capacity to buffer iron is already compromised [218].

However, patient cohorts show heterogeneity: only subsets of septic or critically ill patients display clear systemic ferroptosis signatures, arguing for biomarker-guided stratification in future trials [209].

Direct experimental evidence tying classical cuproptosis (as defined by Tsvetkov) to viral infections [8] is currently limited, but bioinformatic and translational studies indicate dysregulation of cuproptosis-related genes and copper handling in septic shock and other infection contexts, suggesting this pathway may influence infection outcomes (for example, via mitochondrial dysfunction, inflammation amplification or altered immune-cell metabolism) [210,219].

Importantly, cuproptosis biology is tightly coupled to mitochondrial respiration and to cellular metabolic state, features that are often altered during severe infection and with aging—making aged tissues plausibly more susceptible to copper-dependent mitochondrial proteotoxicity when copper handling is disturbed.

#### 5.4.1. Aging and Infection-Related Ferroptotic and Cuproptotic Vulnerability

Several age-associated changes synergistically increase the likelihood that infections will precipitate ferroptotic or cuproptotic injury: (i) Iron dyshomeostasis (tissue accumulation and labile iron increases) elevates the substrate for ferroptosis and lipid peroxidation; (ii) Diminished antioxidant capacity (e.g., lowered GSH/GPX4 activity) reduces the cellular threshold for lipid-peroxide-mediated death; (iii) Mitochondrial decline and proteostasis impairment sensitize cells to the proteotoxic stresses that underlie cuproptosis; and (iv) Immunosenescence and inflammaging promote dysregulated metal redistribution (via altered hepcidin) and reduce resilience to regulated-cell-death-mediated injury [88,220,221]. These converging factors help explain why older adults more frequently progress from infection to severe organ dysfunction in which ferroptosis- and (potentially) cuproptosis-related mechanisms contribute.

#### 5.4.2. Clinical and Research Implications

Available cohort data indicate substantial inter-individual heterogeneity in ferroptosis–related and broader cell-death signatures in older or medically complex patients [209,222,223,224]. A central driver of this variability is the altered hepcidin response that frequently accompanies aging, chronic inflammation and acute systemic stress. Indeed, cohort studies in elderly populations (InCHIANTI, Leiden 85-Plus) have documented that hepcidin correlates with inflammatory markers such as IL-6 or CRP in older individuals [224,225]. Chronic, low-grade inflammation (‘inflammaging’) is similarly linked to elevated hepcidin expression in older adults [226]. Moreover, mechanistic animal models show that IL-6 signalling upregulates hepcidin in aged mice [227]. Geriatric clinical samples also confirm higher hepcidin in chronic disease states [228]. Hepcidin—induced predominantly by IL-6 and related cytokines—downregulates the iron exporter ferroportin, trapping iron within macrophages, hepatocytes and epithelial cells [229]. When hepcidin is chronically elevated or dysregulated, intracellular iron cannot be efficiently exported and accumulates within ferritin and non-ferritin compartments. Under inflammatory or oxidative pressure, ferritin stores undergo ferritinophagy, releasing labile/catalytic iron (Fe^2+^), which readily participates in Fenton chemistry, generating hydroxyl radicals and triggering lipid peroxidation—a hallmark of ferroptosis [230,231]. Thus, dysregulated hepcidin–ferroportin signalling directly enlarges the intracellular labile iron pool (LIP), amplifying susceptibility to ferroptotic death in tissues with high metabolic load (heart, kidney, lung) and in immune cells with high ROS turnover. Cohort studies in critical illness, liver disease, and severe infections show that increased catalytic iron strongly correlates with oxidative damage markers, organ dysfunction, and mortality [232].

For these reasons, pragmatic clinical trials evaluating ferroptosis-targeted or iron-modifying interventions should incorporate biomarkers reflecting both systemic and intracellular iron dysregulation—including serum hepcidin, catalytic/labile iron, lipid-peroxidation products (MDA, 4-HNE), and transcriptional signatures of canonical ferroptosis regulators—to identify patients most likely to benefit from targeted therapies.

Therapeutic strategies aimed at ferroptosis and metal dyshomeostasis (particularly iron and copper) are rapidly emerging as promising approaches in the management of infectious diseases, including severe viral infections and sepsis.

Preclinical models support the idea that inhibiting ferroptosis—or modulating iron—can ameliorate tissue damage in infection. For example, in bacterial and polymicrobial sepsis models (e.g., cecal ligation and puncture), markers of ferroptosis (lipid peroxidation, mitochondrial changes, loss of GPX4 activity) are elevated, and ferroptosis inhibitors (like Fer-1) reduce organ injury. In addition, clinical observations link iron-homeostasis biomarkers (e.g., ferritin, transferrin saturation) to outcomes in septic patients: elevated ferritin (reflecting iron overload) and altered transferrin parameters are associated with worse survival [217,233]

The clinical translation of ferroptosis- and cuproptosis-targeted therapies in infectious diseases faces several important challenges. First, mechanistic infection models are needed to establish causal links between cell-death pathways and pathogen-induced pathology. These models should include both genetic approaches, such as knock-out or knock-down of key regulators (e.g., GPX4 for ferroptosis, FDX1 or DLAT for cuproptosis), and pharmacological modulation using specific inhibitors or metal chelators.

Second, longitudinal human studies are essential to clarify the relationship between metal-homeostasis biomarkers and clinical outcomes. Measuring parameters such as serum iron, ferritin, transferrin, copper, and ceruloplasmin, alongside cell-death signatures (e.g., lipid peroxidation, aggregated lipoylated proteins), in patients across different age groups will help determine how dysregulated metal metabolism contributes to disease severity and age-related susceptibility.

Third, early-phase clinical trials should adopt biomarker-guided enrolment to identify patient subpopulations most likely to benefit from intervention. Ferroptosis inhibitors, copper chelators, or modulators of metal transport could be tested, with both safety and mechanistic endpoints monitored, including metal levels, oxidative stress markers, and inflammatory mediators.

Finally, careful consideration of potential risks is required, since iron and copper are essential for normal cellular function. Therapeutic modulation must be precisely controlled to avoid adverse effects on mitochondrial function, enzymatic activity, or immune responses. Together, these approaches will provide a rational framework for translating metal-dependent cell-death modulation into effective therapies for infectious diseases.

### 5.5. Osteoarticular Diseases

Osteoarticular diseases are the most common chronic degenerative bone diseases with the tissues, structure and functions of bones or joints affection. With age, bone tissue metabolism gradually declines, which disrupts the balance of bone metabolism and causes a number of bone and joint disorders, seriously affecting the patient’s quality of life. As the population ages, the number of people suffering from these diseases is increasing year by year, and the prevalence of bone and joint diseases in the elderly is 27.3% of the elderly population [234].

Osteoarticular diseases (such as osteoarthritis and osteoporosis) are characterized by disrupted homeostasis in bone and joint tissues, with mitochondrial dysfunction, oxidative stress, and dysregulated metal ion metabolism playing prominent roles. Recent mechanistic insights have implicated two regulated cell-death pathways—ferroptosis (iron-dependent lipid peroxidation) and cuproptosis (copper-dependent mitochondrial protein aggregation)—as potentially important drivers of pathology in osteoarticular tissues.

Ferroptosis has been demonstrated in human osteoarthritic (OA) chondrocytes: when chondrocytes are treated with IL-1β, they show accumulation of intracellular iron, increased ROS, MDA, and changes in key ferroptosis proteins; these effects can be reversed by Fer-1, a canonical ferroptosis inhibitor [235]. Moreover, in human OA cartilage, GPX4 (a central ferroptosis regulator) is significantly down-regulated compared to undamaged cartilage, and pharmacologic (Fer-1) or iron chelation (deferoxamine) interventions protect cartilage in vitro and in vivo [235,236]. In addition, the severity of ferroptotic markers in human OA cartilage correlates with disease progression: in patient samples, regions of damaged cartilage (“severe OA”) show more GPX4 down-regulation, ACSL4 up-regulation, mitochondrial depolarization, and lipid peroxidation; importantly, Fer-1 restores chondrocyte viability and increases expression of collagen II (COL2A1) in mild OA, but has less effect in more advanced disease [237].

Multiple studies point to the SLC7A11/GPX4 axis as a key regulatory module: for example, miR-181b is upregulated in OA and can suppress SLC7A11, leading to decreased GPX4 expression and enhanced ferroptotic death of chondrocytes [238]. Furthermore, certain pharmacological agents can prevent ferroptosis: Icariin, a natural flavonoid, protects chondrocytes by upregulating SLC7A11 and GPX4, and reduces OA progression in a rat model [239]. Another regulatory mechanism involves nuclear receptor: peroxisome proliferator-activated receptor gamma (PPARγ). Activation of PPARγ attenuates ferroptosis (induced by RSL3) in rat chondrocytes, likely via enhancement of mitophagy achieved by promoting the Pink1/Parkin-dependent pathway [240]. Genetically, disruption of SCD1 (stearoyl-CoA desaturase-1) exacerbates OA in vivo; SCD1-knockout mice show reduced GPX4 expression, elevated p53, and morphological features of ferroptosis (e.g., shrunken mitochondria) in chondrocytes. Elevated p53 promotes ferroptosis by repressing SLC7A11, which decreases cystine uptake, depletes GSH, and sensitizes chondrocytes to lipid peroxidation [241]. Additionally, under conditions of iron overload, paeoniflorin mitigates OA by reducing ferroptosis via the p53/SLC7A11/GPX4 pathway [242].

These observations strongly support the hypothesis that ferroptosis contributes to chondrocyte death, extracellular matrix (ECM) degradation, and the progression of OA [235]. Moreover, peroxidation of lipids (e.g., MDA, 4-hydroxynonenal) has been identified as a salient feature in OA pathogenesis, linking oxidative stress, ferroptosis, and joint degeneration [243]. Therapeutically, this suggests that interventions aimed at inhibiting ferroptosis—for example, using lipid-peroxidation inhibitors, iron chelators, or agents that restore GPX4/SLC7A11 function—may offer disease-modifying potential in early OA, especially before extensive cartilage damage is established. Indeed, timing appears critical: anti-ferroptotic treatment (e.g., Fer-1) was most effective in chondrocytes from mild OA rather than moderate-to-severe disease [237].

Is noted above, is triggered by intracellular accumulation of copper (Cu^+) and involves aggregation of lipoacylated mitochondrial enzymes of the TCA cycle, including DLAT and DLST, leading to proteotoxic stress and mitochondrial dysfunction [244]. In the context of osteoarticular tissues, dysregulated copper homeostasis has been documented in OA cartilage and synovial fluid, correlating with ECM degradation and chondrocyte dysfunction [39]. Mechanistically, excess intracellular copper induces oligomerization of lipoacylated proteins, destabilization of Fe-S cluster-containing proteins, and activation of proteotoxic stress, ultimately leading to chondrocyte death via cuproptosis [245].

The microenvironment of osteoarticular tissues further modulates cuproptosis. Hypoxia, commonly observed in OA cartilage, affects the expression of copper transporters (e.g., SLC31A1, ATP7B), promoting intracellular copper accumulation and sensitizing chondrocytes to cuproptosis. Stabilization of hypoxia-inducible factor 1-alpha (HIF-1α) appears protective, reducing susceptibility to copper-induced cell death, while inhibition of HIF-1α increases chondrocyte vulnerability [246]. These findings suggest that cuproptosis is not only mechanistically relevant but also therapeutically targetable in osteoarticular diseases.

Historically, the copper chelator D-penicillamine has been used in RA and was shown to inhibit T-cell proliferation in vitro through copper- or ceruloplasmin-dependent hydrogen peroxide generation, suggesting that its immunomodulatory effects may partially reflect modulation of copper metabolism; currently D-penicillamine is considered a second-line treatment [247]. Interestingly, Yu et al. demonstrated that tetrathiomolybdate ammonium (TTM) treatment enhances intracellular GSH levels, stabilizes mitochondrial function and reduces cuproptotic stress, thereby ameliorating OA-related chondrocyte degeneration. Notably, TTM achieved these effects by chelating excess copper, preventing its interaction with lipoylated TCA-cycle proteins, restoring mitochondrial membrane potential, and attenuating ROS accumulation, ultimately preserving chondrocyte viability and slowing OA-associated cartilage damage [39].

In OA models, cuprorivaite-based microspheres suppressed markers of cuproptosis and oxidative stress, reduced histopathological cartilage damage and preserved extracellular matrix components via activation of the Wnt/β-catenin pathway [248]. Additionally, metallic-ion–loaded biomaterials, including those incorporating copper, can modulate exosomal signalling from MSCs and macrophages, thereby influencing inflammation, tissue repair and regeneration [249,250].

Importantly, cuproptosis may intersect with ferroptosis. Excess copper can potentiate oxidative stress, deplete intracellular GSH, and impair GPX4 activity, thereby facilitating ferroptotic cell death in osteoarticular tissues. This cross-talk suggests that copper and iron dysregulation may synergistically contribute to chondrocyte and osteoblast death, accelerating tissue degeneration [39,251].

Therapeutically, targeting both cuproptosis and ferroptosis may offer novel strategies to mitigate osteoarticular tissue degeneration. Potential interventions include copper chelators, modulators of copper transport and lipoacylation, antioxidants, and ferroptosis inhibitors, alone or in combination. Despite promising preclinical data, comprehensive clinical studies are needed to evaluate the safety and efficacy of such strategies, given the essential roles of copper and iron in normal cellular function.

In summary, cuproptosis represents a novel and mechanistically distinct pathway of cell death in osteoarticular diseases. Its interaction with ferroptosis highlights a potential convergent mechanism by which metal dysregulation, oxidative stress, and mitochondrial dysfunction contribute to cartilage and bone degeneration. Further elucidation of these pathways may open avenues for targeted therapies to prevent or slow the progression of OA, osteoporosis, and related disorders.

## 6. Therapeutic Strategies of Ferroptosis and Cuproptosis for Aging and Age-Related Diseases

Targeting ferroptosis and cuproptosis offers a promising therapeutic avenue for mitigating aging and age-related diseases, including neurodegenerative disorders, cardiovascular decline, and chronic inflammatory states.

### 6.1. Pharmacological Modulation

Available cohort data indicate patient heterogeneity in ferroptosis/cell-death signatures; pragmatic trials should include biomarkers of iron catalysis (catalytic iron/labile iron), lipid-peroxidation products and relevant gene-signatures to identify those most likely to benefit from targeted interventions.

The terminal effector of ferroptosis is the uncontrolled accumulation of lipid hydroperoxides, which perpetuate a radical chain reaction across membrane phospholipids, leading to autocatalytic membrane damage and eventual cell rupture. Mechanistically, this process begins via initiation, in which ROS or iron-catalysed Fenton chemistry abstracts a hydrogen atom from bis-allylic positions of polyunsaturated fatty acyl chains, generating a carbon-centred lipid radical (L•) (initiation). This radical reacts rapidly with oxygen to form a lipid peroxyl radical (LOO•), which then propagates the chain reaction by abstracting a hydrogen atom from a neighbouring lipid molecule, forming a new lipid radical and a lipid hydroperoxide (LOOH)—thereby amplifying the radical burden in a self-sustaining cycle [252]. The perpetuation phase can continue until the radicals are neutralized in the termination step, where radical-trapping antioxidants (RTAs) donate a hydrogen atom to LOO•, forming stable non-radical products [26].

Small molecules such as Fer-1 and Lip-1 inhibit ferroptosis by interrupting this chain reaction. They act as high-potency radical-trapping antioxidants (RTAs): Fer-1 and Lip-1 can scavenge peroxyl radicals and even alkoxyl radicals generated by the one-electron reduction of LOOH (driven by ferrous iron), thereby halting the chain propagation. Importantly, their anti-ferroptotic activity is not due to inhibition of lipoxygenase enzymes, but rather to their chemical reactivity with lipid radicals in membranes, breaking the chain reaction before it becomes destructive [26,253]. In fact, Fer-1 has been shown to bind to the 15-lipoxygenase (15-LOX)/phosphatidylethanolamine (PE)-binding protein 1 (PEBP1) complex—a known enzymatic source of peroxidized phospholipids (such as hydroperoxy-eicosatetraenoyl-phosphatidylethanolamine)—and block formation of these death-inducing lipids, further preventing ferroptotic cell death [254].

Fer-1 and Lip-1 efficacy has been validated in multiple preclinical models of aging-related pathology: Fer-1 exerts neuroprotective effects by attenuating ferroptosis in stressed neurons, while Lip-1 has been shown to reduce ischemia–reperfusion injury in the kidney and heart by lowering lipid peroxidation and maintaining tissue viability [255,256]. Notably, in a murine model of isoflurane-induced perioperative neurocognitive disorder, Lip-1 alleviated cognitive decline, restored neuronal and mitochondrial structure, and normalized ferroptosis-related markers of oxidative stress and inflammation, underscoring its potential as a neuroprotective therapy in diverse age-related conditions [257]. Beyond direct ferroptosis inhibition, modulation of systemic iron burden provides an additional therapeutic axis, as excessive brain iron accumulation is a hallmark of aging and contributes to the pathogenesis of AD and PD. Iron chelators such as deferiprone and deferoxamine have demonstrated neuroprotective effects in both clinical and preclinical contexts, with deferiprone reducing neuronal loss and improving motor function in PD models [258], and deferoxamine attenuating amyloid plaque deposition and oxidative injury in AD models [250,259,260]. Collectively, these findings highlight both ferroptosis inhibitors and iron chelators as convergent strategies to counteract lipid peroxidation and iron dysregulation, thereby addressing fundamental drivers of neurodegeneration and functional decline during aging.

In cuproptosis, therapeutic development remains nascent, yet insights from copper dysregulation disorders provide a foundation. Copper chelators such as tetrathiomolybdate and penicillamine, used clinically in Wilson’s disease (WD), reduce systemic copper by forming excretable complexes or enhancing urinary clearance, thereby lowering mitochondrial oxidative stress and protecting against age-associated tissue damage [261]. Conversely, copper ionophores like elesclomol import copper into mitochondria, where accumulation triggers lipoylation-dependent protein aggregation, destabilization of Fe-S clusters, and selective death of metabolically vulnerable tumour cells—an approach relevant for oncological diseases in aging populations [262]. This dual paradigm underscores the copper paradox, where copper is indispensable for mitochondrial respiration and antioxidant enzyme function but cytotoxic when misregulated, particularly in the context of aging, mitochondrial dysfunction, and inflammaging [8].

Emerging precision medicine strategies aim to optimize ferroptosis- and cuproptosis-targeted interventions through biomarker-guided approaches, employing indicators such as serum ferritin, transferrin saturation, labile plasma iron, copper/zinc ratios, GPX4 activity, and lipid peroxidation products to identify individuals most susceptible to metal-driven cell death [263,264,265]. Nanomedicine platforms, including liposomal ferroptosis inhibitors and organ-specific chelators, are under development to deliver therapies with enhanced tissue selectivity (e.g., brain, heart, kidney) while minimizing systemic toxicity [266]. Parallel strategies aimed at restoring the aging microenvironment—specifically, senolytic ablation of senescent cells coupled with immunomodulatory agents that restore efferocytosis and suppress NLRP3 inflammasome activity—offer complementary mechanisms for intervention. Concomitant modulation of regulated cell death pathways, such as ferroptosis and cuproptosis, may synergistically potentiate these approaches to attenuate SASP-driven inflammaging and chronic tissue degeneration. Efferocytosis, the process by which phagocytes recognize, engulf, and clear apoptotic cells, is essential for maintaining tissue homeostasis and resolving inflammation [267,268]. In the context of aging, this crucial “silent” clearance mechanism often becomes impaired, leading to a build-up of uncleared apoptotic cells, which can undergo secondary necrosis and release pro-inflammatory DAMPs [269]. Restoring efficient efferocytosis is therefore a vital immunomodulatory strategy to prevent chronic inflammation and promote a pro-resolving tissue microenvironment following the removal of senescent cells or other damaged cellular components [270,271,272]. At the mechanistic core, the GSH axis represents the principal endogenous defence against ferroptosis, with GPX4 detoxifying lipid hydroperoxides to prevent membrane damage [273]. Age-associated depletion of intracellular GSH impairs this protective system, predisposing cells to oxidative stress and ferroptotic death [264,274]. N-acetylcysteine (NAC) and related cysteine donors replenish GSH synthesis, thereby sustaining GPX4 activity and reinforcing cellular resistance to lipid peroxidation. Preclinical evidence demonstrates that NAC supplementation restores redox homeostasis and attenuates oxidative injury across multiple models of aging and neurodegeneration, and early-phase clinical studies in PD and AD have reported improvements in antioxidant status, mitochondrial function, and, in some cases, cognitive or dopaminergic outcomes [275,276,277].

### 6.2. Natural Modulation of Ferroptosis and Cuproptosis

**Figure 2:**
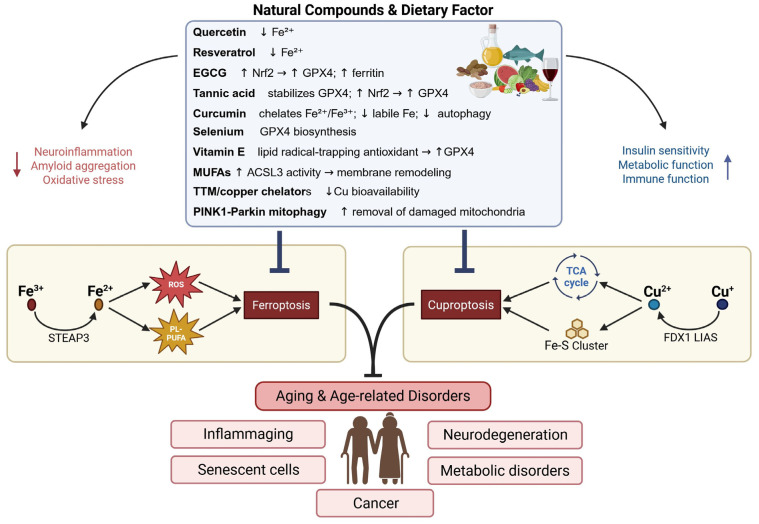
***Natural compounds and dietary factors modulate ferroptosis and cuproptosis pathways implicated in aging.*** Bioactive molecules reduce Fe^2+^/Cu^2+^ availability, enhance GPX4 stability or biosynthesis, activate Nrf2 signalling, remodel membranes, or promote mitophagy, thereby suppressing lipid peroxidation–driven ferroptosis and copper-dependent protein lipoylation stress underlying cuproptosis. By attenuating these regulated cell death pathways, such interventions mitigate neuroinflammation, oxidative stress, and metabolic decline, ultimately slowing inflammaging and reducing susceptibility to age-related disorders including neurodegeneration, metabolic dysfunction, and cancer. Abbreviations: ACSL3, acyl-CoA synthetase long-chain family member 3; FDX1, ferredoxin 1; Fe-S, iron-sulphur; GPX4, glutathione peroxidase 4; LIAS, lipoic acid synthase; NRF2, nuclear factor erythroid 2-related factor 2; PL-PUFA, phospholipid polyunsaturated fatty acids; ROS, reactive oxygen species; STEAP3, six-transmembrane epithelial antigen of the prostate 3; TCA, tricarboxylic acid cycle (Krebs cycle); TTM, tetrathiomolybdate. Figure created with BioRender.com (accessed on 12 December 2025).

Natural dietary components, phytochemicals, and lifestyle interventions modulate susceptibility to metal-dependent RCD—most prominently ferroptosis, and, through effects on metal homeostasis and mitochondrial proteostasis, pathways relevant to cuproptosis—at multiple mechanistic levels (Figure 2).

A broad range of natural compounds and dietary factors influence metal homeostasis and thereby modulate ferroptotic vulnerability. Among them, plant polyphenols—including quercetin, resveratrol, curcumin and epigallocatechin-3-gallate (EGCG)—exert multi-level protective actions against ferroptosis and metal-driven oxidative injury. These compounds display a characteristic triad of biological effects:

(i) Direct radical-scavenging, which neutralizes lipid peroxyl radicals and interrupts lipid peroxidation chains;
(ii) Transition-metal chelation, lowering labile Fe^2+^ and Cu^+^ pools that catalyse Fenton-type reactions;
(iii) Activation of adaptive transcriptional programs, particularly the NRF2–ARE axis, which promotes GSH synthesis and GPX4-mediated detoxification of lipid hydroperoxides [88].

Experimental studies illustrate these mechanisms. Quercetin and resveratrol suppress erastin- and RSL3-induced ferroptosis in HT22 neuronal cells by attenuating ROS levels and reducing the intracellular labile iron pool independently of NRF2 activation [278]. Similarly, EGCG inhibits ferroptosis in vivo in a mouse model of colitis by activating the Nrf2–GPX4 pathway and enhancing ferritin expression in colonic epithelial cells [279]. Additional evidence shows that EGCG mitigates iron deposition and oxidative stress in intracerebral haemorrhage through activation of Keap1/p62/Nrf2 signalling, leading to in-creased GPX4 and system Xc^−^ expression [280].

Curcumin further exemplifies the combined antioxidant and metal-binding properties of polyphenols. It forms stable complexes with Fe^2+^/Fe^3+^, lowering free iron levels and suppressing iron-induced autophagy and cell death in hepatic and epithelial cell models [281]. Engineered curcumin–polydopamine nanoparticles demonstrate enhanced iron-chelating capacity, reduce Fe^2+^ accumulation, and protect against ferroptotic lipid peroxidation in the PC12 neuronal cell line [282]. Beyond iron modulation, curcumin also interacts with copper: biochemical and in vivo studies indicate that curcumin forms complexes with copper and alters tissue copper distribution, providing a plausible mechanism for modulating copper-dependent proteotoxic stress implicated in cuproptosis [283,284].

Collectively, the evidence supports that polyphenols can modulate ferroptosis and potentially cuproptosis through a combination of direct antioxidant activity, metal se-questration, and NRF2-GPX4 pathway activation. However, factors such as bioavailabil-ity, metabolic conversion, and tissue distribution should be considered when translating these findings to clinical settings [88,283,284]

Micronutrients exert a profound influence on physiological function; notably, selenium is indispensable for the biosynthesis of the selenoprotein GPX4, a key suppressor of ferroptosis [285]. Deficiency of selenium or modulation of GPX4 expression may dramatically affect the redox buffering capacity against lipid peroxidation and ferroptosis. Meanwhile, vitamin E (α-tocopherol) works synergistically with GPX4: studies in hematopoietic progenitor cells have shown that α-tocopherol rescues GPX4-deficient cells from ferroptosis, highlighting the cooperative defence against lipid peroxidation [286]. Moreover, vitamin E (as a lipophilic radical-trapping antioxidant) is increasingly recognized as a frontline defence against ferroptosis in many systems [100]. On the copper side, copper homeostasis and its disturbance are increasingly relevant via cuproptosis, a copper-dependent form of cell death. Dietary or endogenous copper overload can promote intracellular accumulation, and copper chelators such as TTM can reduce copper bioavailability and thus suppress cuproptosis [287]. For instance, in cancer contexts, TTM and related chelators are discussed for their ability to inhibit copper-driven proteotoxic stress linked to cuproptosis [162].

Beyond their antioxidant and iron-chelating actions, natural polyphenols can directly modulate ferroptosis effectors. Tannic acid has been reported to bind GPX4 at a non-inhibitory allosteric pocket, stabilizing the enzyme’s active conformation and increasing its rate of lipid hydroperoxide reduction. Concurrently, tannic acid activates Nrf2 signalling, leading to transcriptional upregulation and accumulation of GPX4 protein in cells, thereby establishing a dual mechanism—enzyme activation plus enhanced expression—to suppress ferroptotic lipid peroxidation [288].

Dietary fatty-acid composition directly modulates ferroptotic sensitivity. PUFAs increase the abundance of ACSL4-activated, lysophosphatidylcholine acyltransferase 3 (LPCAT3)–assembled PUFA-phospholipids that serve as substrates for both enzymatic and radical-driven lipid peroxidation, thereby promoting ferroptosis. In contrast, monounsaturated fatty acids (MUFAs) attenuate ferroptosis by engaging acyl-CoA synthetase long-chain family member 3 (ACSL3), diverting acyl-CoA flux away from PUFA incorporation and remodelling membranes with oxidation-resistant species. Oleic-acid supplementation was shown to confer potent, ACSL3-dependent ferroptosis protection, establishing a defined metabolic mechanism linking fatty-acid availability to ferroptosis thresholds [289]. Mitochondrial quality control represents a second major axis through which diet and lifestyle alter metal-dependent regulated cell death. Selective removal of dysfunctional mitochondria by mitophagy (for example via the PTEN-induced kinase 1 (PINK1)–Parkin pathway) prevents accumulation of mitochondria that generate excess reactive oxygen species and display impaired iron–sulphur cluster handling—conditions that amplify lipid peroxidation and lower ferroptotic thresholds [290]. These mechanistic intersections are particularly pertinent to aging, where inflammaging, accumulation of senescent cells and dysregulated metallostasis converge. Multiple studies report that senescent cells accumulate iron, display altered ferritinophagy and lysosomal dysfunction, and thereby perturb cellular iron handling and oxidative balance—changes that reshape ferroptosis responsiveness in complex ways [109]. Senescent cell survival and SASP signalling further modify tissue iron handling and inflammatory milieu, linking senescence biology to metal-driven redox pathology [291].

**Figure 3:**
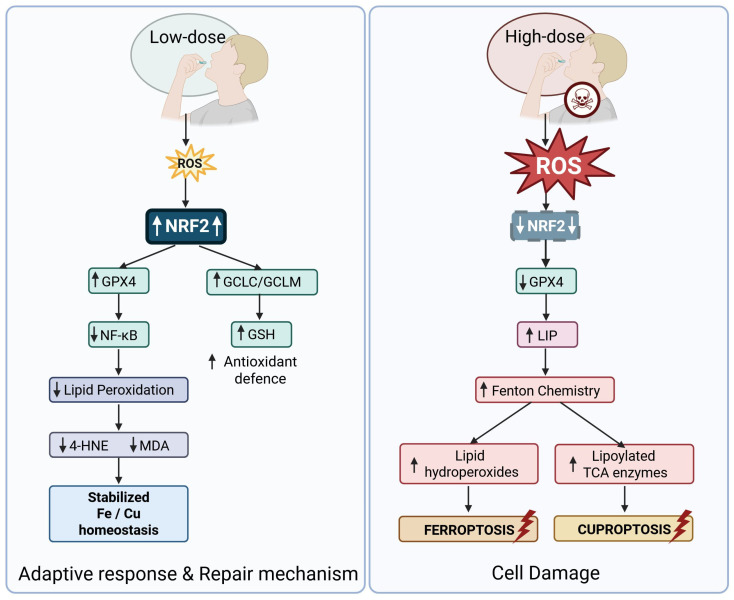
***Hormesis-driven, dose-dependent ROS signalling differentially regulates NRF2 activity and vulnerability to ferroptosis and cuproptosis.*** Low ROS levels induce a hormetic NRF2 activation response that upregulates GPX4 and GCLC/GCLM, boosts GSH synthesis, suppresses NF-κB signalling, and limits lipid peroxidation, thereby lowering 4-HNE and MDA formation and stabilizing Fe/Cu homeostasis. In contrast, high ROS levels overwhelm the hormetic window, suppress NRF2 activity, reduce GPX4, increase the labile iron pool, and intensify Fenton chemistry, leading to lipid hydroperoxide buildup and aberrant lipoylation of TCA-cycle enzymes. These conditions promote ferroptosis and cuproptosis, culminating in oxidative cell damage. Abbreviations: 4-HNE, 4-hydroxynonenal; GCLC/GCLM, glutamate-cysteine ligase catalytic subunit/glutamate-cysteine ligase modifier subunit; GPX4, glutathione peroxidase 4; GSH, glutathione; LIP, labile iron pool; MDA, malondialdehyde; NF-κB, nuclear factor kappa-light-chain-enhancer of activated B cells; NRF2, nuclear factor erythroid 2-related factor 2; ROS, reactive oxygen species; TCA, tricarboxylic acid cycle (Krebs cycle). Figure created with BioRender.com (accessed on 12 December 2025).

In addition to their direct antioxidant and metal-modulating actions, many natural dietary compounds operate according to the principle of hormesis—a biphasic, dose-dependent adaptive response in which low or moderate exposure to a stressor induces beneficial cellular defences, whereas high doses are detrimental (Figure 3).

This concept is increasingly recognized as central to understanding how polyphenols, flavonoids, terpenoids, and other nutraceuticals modulate ferroptosis and cuproptosis pathways. At low physiological concentrations, these “hormetic nutrients” activate cytoprotective signalling cascades such as the Nrf2–GSH–GPX4 axis and suppress pro-inflammatory pathways including NF-κB, thereby enhancing antioxidant capacity, restoring redox resilience, and delaying aging-related ferroptotic/cuproptotic vulnerability [292,293,294,295]. Experimental studies demonstrate that low-dose administration of hormetic compounds upregulates Nrf2, increases intracellular GSH pools, and elevates GPX4 activity, collectively blocking lipid peroxidation and preventing ferroptotic cell death in neuronal and metabolic aging models [292,293,294]. Conversely, high doses of the same compounds can become pro-oxidant, inhibit antioxidant enzymes, and promote iron- or copper-dependent lipid peroxidation, ultimately facilitating ferroptosis or cuproptosis and contributing to chronic diseases [293,296,297]. This duality highlights the importance of dose-optimization as a core principle of hormetic healing medicine.

To provide a clearer overview of how specific natural compounds exert dose-dependent hormetic effects on ferroptosis and cuproptosis pathways, we compiled key examples in Table 1. The table summarizes representative “hormetic nutrients” that have been experimentally shown to modulate redox homeostasis and metal-dependent cell-death mechanisms through dual actions. The table also includes references to specific experimental studies (PMIDs provided), highlighting the evidence base for each compound across various disease models, including neurodegeneration, metabolic aging, and cancer. By summarizing these hormetic patterns, Table 1 illustrates the importance of dose as a critical determinant of whether natural compounds confer cytoprotection or promote cell death, reinforcing the concept of hormesis as a guiding principle in nutritional and pharmacological modulation of ferroptosis and cuproptosis.

#### 6.2.1. Polyphenols: Combined Radical Scavenging, Metal Binding and Nrf2 Induction

Plant polyphenols (for example curcumin, quercetin, resveratrol, epigallocatechin gallate and proanthocyanidins) display a triad of actions relevant to ferroptosis and metal toxicity: (i) direct radical-scavenging that neutralizes lipid radicals and halts chain peroxidation; (ii) ability to chelate transition metals and thereby lower the labile Fe^2+^/Cu^+^ pools that catalyse Fenton chemistry; and (iii) activation of adaptive transcriptional programmes (notably the Nrf2–antioxidant response element (ARE) axis) that up-regulate cytoprotective genes including those involved in GSH synthesis and GPX4-dependent lipid repair [88]. Comprehensive reviews and mechanistic studies support the capacity of many polyphenols to attenuate lipid peroxidation and to modulate ferroptosis-related endpoints in cellular and animal models, although bioavailability and in vivo metabolism must be considered when extrapolating to clinical settings [88,283,302].

Curcumin exemplifies a dual antioxidant/metal-binding natural product: biochemical and in vivo data show curcumin complexes with copper and can alter copper-dependent signalling and tissue copper content, providing a plausible mechanistic link to modulation of copper-driven proteostatic stress that underlies cuproptosis-related biology [283,284].

#### 6.2.2. Dietary Trace-Metal Modulation and Phytate Effects

Population-level and interventional data indicate that dietary composition markedly affects systemic iron bioavailability. Phytate (myo-inositol hexaphosphate), abundant in legumes, nuts, and whole grains, is a strong chelator of divalent metal ions and forms insoluble complexes with non-heme iron in the intestinal lumen. By reducing the solubility of iron at physiological pH, phytate decreases the fraction of iron available for absorption by the enterocyte divalent metal transporter 1 (DMT1). Consequently, phytate modulates postprandial iron kinetics, attenuating the rise in circulating serum iron and transferrin saturation that normally occurs after a meal [303,304,305,306,307].

Controlled human feeding studies using radioisotopic tracers (^55^Fe, ^59^Fe) have demonstrated that phytate can substantially inhibit non-heme iron absorption, with the magnitude of inhibition dependent on the dose of phytate and the composition of the meal. For example, Hallberg et al. [303] showed that increasing doses of phytate in test meals significantly reduced iron absorption in healthy volunteers, while Brune et al. [304] demonstrated that chronic consumption of high-phytate diets does not trigger intestinal adaptation, confirming the persistent inhibitory effect of phytate on iron uptake [303,304].

These findings support dietary modulation of phytate intake as a practical approach to limit the systemic pool of labile iron and reduce susceptibility to iron-driven lipid peroxidation and ferroptotic stress.

Similarly, avoiding unnecessary copper supplementation in older adults who may already have elevated copper stores is a prudent, biomarker-guided measure: experimental data in cell and animal models show that copper overload can impair mitochondrial function, induce oxidative stress, and provoke proteotoxic stress via lipoylation of mitochondrial proteins, suggesting potential risks of intracellular copper accumulation in vulnerable individuals [308,309,310].

#### 6.2.3. Lifestyle Interventions: Exercise, Mitochondrial Quality Control and Nutrient Signalling

Exercise and caloric restriction exert convergent effects on mitochondrial resilience and antioxidant defences, which may influence cellular susceptibility not only to ferroptosis but also, indirectly, to copper-dependent mitochondrial stress. Early work in rodent models demonstrated that endurance training alters systemic copper handling: physical training increases ceruloplasmin activity and redistributes copper from storage tissues (e.g., liver and myocardium) to circulation, indicating a remodelling of copper homeostasis under sustained metabolic load [311,312]. Such shifts in copper distribution may influence intracellular copper availability and thereby affect pathways related to copper toxicity. Although direct evidence that exercise modulates cuproptosis is currently lacking, several mechanistic links are plausible based on experimental studies. In a mouse model of amyotrophic lateral sclerosis, swim training reduced skeletal-muscle copper accumulation by decreasing CTR1 (copper importer) and increasing ATP7A (copper exporter), suggesting that exercise can actively modify copper transport machinery [313]. Conversely, studies of chronic copper overload in rats show that excessive copper impairs mitochondrial respiration, decreases cytochrome-c oxidase activity, and increases lipid peroxidation [4], illustrating how copper dysregulation directly disrupts mitochondrial function—central to the execution of cuproptosis [314]. In contrast, the protective effects of exercise against ferroptosis are better established. Preclinical studies demonstrate that aerobic training up-regulates the cystine/glutamate antiporter system x_c^−^ and GPX4, reduces iron overload, and limits lipid peroxidation in aging and neurodegenerative models [116]. Similarly, interventions that activate NAD^+^/SIRT signalling and suppress mTOR activity—such as caloric restriction, intermittent fasting, and caloric-restriction mimetics—promote mitophagy, mitochondrial biogenesis, and antioxidant capacity, collectively raising the threshold for ferroptotic cell death in aged tissues [315,316].

Taken together, while robust evidence supports the role of exercise and nutrient-signalling pathways in modulating ferroptotic susceptibility, their influence on cuproptosis remains hypothetical and is likely mediated through broader effects on copper transport, mitochondrial quality control, and redox homeostasis.

#### 6.2.4. Targeted Delivery: Nanomedicine Approaches to Chelation and Redox Modulation

Nanoparticle-based platforms offer improved tissue selectivity and pharmacokinetics for both metal chelators and ferroptosis inhibitors compared with conventional small-molecule chelators. Small-molecule chelators such as deferoxamine (DFO), deferiprone (DFP) and deferasirox (DFX) are low-molecular-weight agents with well-documented clinical efficacy in transfusional iron overload, but they have important limitations: short plasma half-life (particularly for DFO), requirement for parenteral or frequent dosing, dose-limiting systemic toxicities (nephrotoxicity, auditory/visual effects, agranulocytosis for some agents), and limited ability to selectively accumulate in target organs such as the brain or injured myocardium. These properties can constrain chronic use in older adults and reduce organ-specific efficacy [259,317].

Nanochelator and nanoparticle delivery strategies address several of these shortcomings by (i) altering biodistribution to favour accumulation in selected tissues, (ii) improving chelator pharmacokinetics and residence time at the target site, (iii) enabling renal clearance of the chelator–metal complex to minimise reticuloendothelial sequestration, and (iv) reducing peak systemic exposure that drives off-target toxicity. For example, a renal-clearable polymeric nanochelator demonstrated rapid urinary excretion (>80% of the injected dose recovered in urine within hours in mice), kidney-selective biodistribution after single-dose administration, and markedly improved efficacy and safety relative to free DFO in rodent iron-overload models (increased iron excretion, lower hepatic iron, and less nephrotoxicity). These design features translated into greater pharmacodynamic efficiency (several-fold improvement in iron removal per dose) in preclinical studies [318,319,320].

Other nanoparticle approaches use active targeting or biomimetic coatings to deliver chelators to specific lesions. Rabies-virus-glycoprotein (RVG)–modified polymeric nanoparticles loaded with DFO crossed the blood–brain barrier and reduced iron accumulation and behavioural deficits in Parkinsonian mice, demonstrating improved brain delivery compared with free DFO. Platelet-membrane-coated nanoparticles and ligand-functionalized constructs have similarly been used to direct chelators to sites of vascular or cerebral injury, increasing local chelator concentration while lowering systemic exposure [321]. Finally, nanoparticle formulations support combination strategies—for instance, co-delivery of iron chelators with antioxidants or ferroptosis inhibitors—and carrier-free high-loading designs for chronic, organ-targeted therapy. For example, RVG29-modified nanoparticles loaded with deferoxamine (DFO) cross the blood–brain barrier and reduce iron deposition and oxidative stress in a PD mouse model [321].

Macrophage-membrane coated nanoparticles functionalized with RVG29 and thiamin pyrophosphate (TPP) deliver genistein to neuronal mitochondria, combining antioxidant action with efficient targeting [322]. In a different design, ROS-responsive nanoparticles encapsulating curcumin release their payload in oxidative microenvironments, thereby enhancing antioxidant capacity and minimizing systemic exposure [323]. Moreover, renal-clearable nanochelators bearing DFO show >80% urinary excretion, tissue-selective iron removal, and significantly reduced off-target toxicity compared to free DFO in animal models [318]. Taken together, preclinical evidence supports a translational path whereby nanochelators provide improved organ targeting, enhanced iron excretion efficiency, and reduced systemic toxicity compared with conventional small-molecule chelators.

#### 6.2.5. Repleting the GSH–GPX4 Axis: Cysteine Donors and Translational Evidence

The GSH–GPX4 axis is the central enzymatic defence against lipid peroxidation and ferroptosis. Nutritional or pharmacological replenishment of cysteine and GSH (for example with NAC) restores brain and systemic GSH pools in human studies and improves biochemical indices of oxidative buffering in neurodegenerative cohorts, supporting the concept that targeted support of thiol metabolism can mitigate ferroptosis-relevant vulnerability in aging tissues [324,325,326,327]. Controlled, disease-specific trials with ferroptosis-relevant end points are still required to define clinical efficacy and optimal dosing regimens.

#### 6.2.6. Modulating the Aging Microenvironment: Senolytics, Efferocytosis and Inflammasome Inhibition

Senescent cells contribute to inflammaging via the SASP, which amplifies oxidative stress and perturbs local metal handling. Senescent cells commonly display mitochondrial dysfunction with increased mitochondrial ROS production and altered NAD^+^/NADH ratios; mitochondrial defects both trigger senescence and sustain a modified, mitochondria-linked SASP that elevates local oxidative burden [328,329].

The SASP itself (IL-1β, IL-6, TNF-α and other mediators) activates NF-κB/JAK–STAT signalling in neighbouring cells, inducing secondary ROS production and a feed-forward inflammatory loop that amplifies tissue oxidative stress [330].

Senescence also disrupts cellular iron handling. Multiple studies show that senescent cells accumulate stored iron (increased ferritin) and exhibit impaired ferritin degradation (ferritinophagy), which expands the labile iron compartment in ageing tissues and promotes Fenton chemistry–driven ROS and lipid peroxidation [109].

Impaired NCOA4-dependent ferritinophagy has recently been identified in senescent fibroblasts and diabetic wound tissue, linking defective ferritin turnover to altered ferroptotic sensitivity and persistent oxidative stress in vivo [331]. Recent work also documents iron accumulation in fibrotic, senescence-rich tissues, further supporting a model in which senescent-cell iron retention fuels local redox toxicity and SASP amplification [332].

Because senescent cells therefore both increase ROS production and alter metal homeostasis, their accumulation creates a microenvironment in which neighbouring cells are more susceptible to iron-driven lipid peroxidation (ferroptosis) and—by analogy—may be more vulnerable to other metal-dependent stresses. Early human studies of senolytic therapy (dasatinib + quercetin) have demonstrated reductions in senescent-cell markers and circulating SASP mediators, providing proof-of-principle that targeting senescent cells can lower tissue-level pro-oxidant signalling and merit further investigation as an adjunct to limit ferroptotic (and possibly cuproptotic) susceptibility. The aging tissue microenvironment is shaped by three mutually reinforcing processes: senescent-cell accumulation, impaired efferocytosis, and chronic activation of innate immune pathways such as the NLRP3 inflammasome. Together, these mechanisms amplify oxidative stress, disturb local metal homeostasis and create conditions that sensitise cells to ferroptotic and cuproptotic injury [330].

Complementary immunomodulatory strategies that restore effective efferocytosis and restrain maladaptive innate immune activation may help normalize redox signalling in aging tissues. Small-molecule NLRP3 inhibitors such as N-[[(\(1,2,3,5,6,7\)-hexahydro-\(s\)-indacen-4-yl)amino]carbonyl]-4-(1-hydroxy-1-methylethyl)-2-furansulfonamide, monosodium salt (MCC950) potently block canonical NLRP3 activation in preclinical models. In aged mice, MCC950 reduces IL-1β/IL-18 maturation, attenuates pyroptotic signalling, and improves metabolic and organ function by limiting chronic inflammasome activity [333,334].

In models of age-related cardiac and neurological dysfunction, MCC950 also decreases oxidative stress markers and cellular senescence, indicating that NLRP3 inhibition can dampen inflammasome-driven amplification of oxidative injury [335]. By suppressing this feed-forward inflammatory–redox loop, NLRP3 inhibitors may indirectly reduce susceptibility to ferroptotic stress in aging microenvironments.

#### 6.2.7. Integrative, Biomarker-Guided Geroprotective Strategies

A clinically useful framework will require validated biomarkers (for example serum ferritin, transferrin saturation, measures of non-transferrin-bound/labile plasma iron, copper/zinc ratio, GPX4 activity and quantitative lipid-peroxidation assays such as 4-HNE/MDA adducts), combined with tissue-targeted delivery platforms and combinatorial regimens pairing diet, exercise, phytochemicals and selective pharmacological agents. Recent reviews synthesise mechanistic links between iron homeostasis and ferroptosis across human diseases, underscoring the translational potential for integrated approaches that reduce metal burden and strengthen antioxidant repair systems in aging [336,337,338].

## 7. Limitations of the Study

Despite the comprehensive scope of this review, several limitations should be acknowledged. First, because ferroptosis and cuproptosis are rapidly evolving research areas, the mechanistic understanding of their interplay during aging remains incomplete. Many conclusions presented here rely on associations described in preclinical models, and definitive causal relationships—particularly the convergence of iron- and copper-dependent death pathways in human aging—have yet to be experimentally validated.

Second, substantial heterogeneity exists across available studies in terms of experimental systems, dosages, animal models, and methodological approaches used to detect regulated cell death, redox markers, or metal dyshomeostasis. This variability limits direct comparability and may bias the interpretation of emerging patterns.

Third, while we emphasize the potential of natural hormetic compounds and nutritional or pharmacological interventions to modulate ferroptosis and cuproptosis, clinical evidence remains scarce. Most of the data derive from in vitro and rodent studies, and their translational relevance to human aging, multimorbidity, and inflammaging is uncertain. Fourth, the literature on cuproptosis is still in an early stage, with few studies extending beyond foundational mechanistic descriptions. As a result, our integrated model connecting ferroptosis, cuproptosis, mitochondrial dysfunction, and inflammaging necessarily incorporates conceptual extrapolations that require further experimental verification.

Finally, despite efforts to include up-to-date and primary experimental research, some reliance on review articles was unavoidable due to gaps in mechanistic studies, particularly in aging contexts. Future work should address these limitations by implementing standardized experimental frameworks, expanding longitudinal human studies, and conducting targeted interventions that directly test the therapeutic potential of modulating metal-regulated cell death pathways in aging.

## 8. Conclusions

The discovery and ongoing characterization of ferroptosis and cuproptosis have significantly expanded our understanding of RCD mechanisms implicated in age-related diseases. While apoptosis and necroptosis have long been central to the paradigm of immune aging and degeneration, ferroptosis and cuproptosis represent previously underappreciated contributors that help bridge the gap between redox imbalance, mitochondrial dysfunction, and immunosenescence. Importantly, the selective vulnerability of certain cell types—such as neurons, hepatocytes, and T cells—to ferroptosis or cuproptosis may help explain the organ-specific manifestations of age-related disorders.

### 8.1. Emerging Biomarkers

The unique biochemical and molecular signatures of ferroptosis and cuproptosis provide tractable biomarker candidates for diagnosis, prognostication and patient stratification in aging-related disease. For ferroptosis, a consistent pattern across cellular, animal and translational studies includes accumulation of lipid peroxidation products (notably 4-HNE, and MDA), loss or functional inhibition of GPX4, and the presence of redox-active non-transferrin-bound (labile) iron species in plasma [189,339,340]. Assays that quantify lipid-peroxidation adducts (4-HNE–protein adducts, MDA- TBARS or more specific liquid chromatography-mass spectrometry (LC–MS) methods) together with measures of GPX4 expression/activity and labile plasma iron (LPI/non-transferrin-bound iron (NTBI) therefore form a rational multimodal panel to capture ferroptotic propensity and systemic iron redox burden [31,37,143].

For cuproptosis, the discovery-phase work implicating FDX1-dependent protein lipoylation and lipoylated TCA-cycle components as the mechanistic nexus identifies candidate molecular markers: increased cellular copper content coupled with elevated protein lipoylation, and altered expression of cuproptosis-regulatory genes such as FDX1 and LIAS may signal cuproptosis-prone states [8,164]. At the clinical level, simple systemic indices—for example the serum copper: zinc ratio—have long been associated with aging, inflammation and adverse outcomes and may serve as an accessible, if non-specific, indicator of altered copper handling that merits further evaluation in cuproptosis-focused studies [341,342].

Complementary biomarker axes include gene-expression signatures and protein-level readouts of canonical ferroptosis regulators (solute carrier family 7 member 11 (SLC7A11), GPX4, FSP1/apoptosis-inducing factor, mitochondrion-associated, 2 (AIFM2), acyl-CoA synthetase long chain family member 4 (ACSL4)) and cuproptosis-linked factors (FDX1, LIAS, components of the lipoylation machinery). Among these, ACSL4 plays a central mechanistic role in ferroptosis: it activates PUFAs like arachidonic acid (AA) and adrenic acid (AdA) by converting them into their CoA derivatives, which are then esterified into phospholipids. These PUFA–phospholipids constitute the preferred substrates for lipid peroxidation that drives ferroptotic membrane damage [343]. Loss or inhibition of ACSL4 has been shown to strongly protect cells from ferroptosis: genetic deletion of ACSL4 renders cells resistant to classical ferroptosis inducers, and pharmacological inhibitors of ACSL4 reduce lipid peroxides and ferroptotic cell death in disease models [344,345]. Additionally, in a mouse model of ischemic stroke, ACSL4 is upregulated after ischemia–reperfusion, and blocking ACSL4 via small-molecule inhibitors (e.g., rosiglitazone) reduces iron accumulation, oxidative stress, and ferroptotic markers, thereby improving neurological recovery [346]. Further, ACSL4 regulation can be influenced by cellular stress pathways: in glioma cells, heat shock protein Hsp90 and dynamin-related protein 1 (Drp1) dephosphorylation stabilize ACSL4 expression during erastin-induced ferroptosis, promoting mitochondrial fragmentation and lipid peroxidation [347]. Given this, ACSL4 not only serves as a biomarker of ferroptotic vulnerability but also represents a drug-gable node for interventions directed at pathological ferroptosis in aging tissues.

Integration of bulk and single-cell transcriptomic datasets enables identification of cell-type-specific transcriptional programs associated with ferroptotic/cuproptotic vulnerability in aging tissues; large single-cell atlases (e.g., Tabula Muris Senis) and immune-focused single-cell studies provide a resource to map these signatures to cell populations that expand or decline with age, and to study co-localization with senescence/inflammaging programs [348,349,350]. Finally, spatial transcriptomics and combined proteomic/lipidomic imaging platforms will be essential to link molecular signatures (lipid peroxidation hotspots, lipoylated mitochondrial protein aggregates, local copper enrichment) to histological patterns of tissue degeneration and SASP-rich microenvironments.

In summary, a pragmatic biomarker strategy for aging-relevant ferroptosis/cuproptosis should combine: (i) direct chemical measures of oxidative lipid damage (4-HNE, MDA, isoprostanes) and labile iron/copper fractions; (ii) activity or abundance assays for core regulators (GPX4, FSP1/AIFM2, SLC7A11, FDX1, LIAS); and (iii) cell-resolved transcriptomic/spatial readouts to map vulnerability to specific cell populations and microenvironments. Prospective studies that correlate multimodal biomarker panels with functional outcomes in aging cohorts are now required to validate these candidates and establish clinically useful thresholds.

### 8.2. Final Remarks

In conclusion, ferroptosis and cuproptosis constitute novel, mechanistically distinct, and clinically relevant links in the aging and inflammaging cascade. Their contributions to tissue degeneration, immune activation, and metabolic imbalance are increasingly recognized, offering new opportunities for biomarker discovery and therapeutic innovation. Future research must continue to map these processes across organs and disease states, while embracing interdisciplinarity to translate molecular insights into clinical impact.

## Figures and Tables

**Table 1:** Hormetic natural compounds and their effects on ferroptosis/cuproptosis pathways.

Compound	Low-Dose Effect (Typical)	High-Dose Effect (Typical)	Selected Experimental Evidence	Disease Models/Contexts
**Polyphenols (general)**	Activation of Nrf2 → ↑ GSH, ↑ GPX4; inhibition of NF-κB; improved redox resilience	Pro-oxidant effects at high concentrations → ↑ ROS, promote lipid peroxidation and RCD	[292,293,295,296]	Neurodegeneration, metabolic ageing, inflammation, cancer (dose-dependent effects)
**Curcumin**	Induces Nrf2 signalling; suppresses NF-κB; raises antioxidant enzyme expression	At high doses: oxidative stress, cytotoxicity in some cell types	[298,299]	Neuroprotection models, metabolic inflammation, cancer cell lines
**Resveratrol**	Enhances mitochondrial resilience, activates Nrf2/SIRT1 cross-talk, supports GSH/GPX4	High doses may increase ROS, trigger cell death in cancer cells	[293,300]	Neurodegeneration, metabolic syndrome, cancer (chemosensitization)
**EGCG (green tea catechin)**	Stimulates antioxidant responses (Nrf2); can increase GPX4 activity indirectly	At supraphysiological doses may act as pro-oxidant and induce apoptosis/ferroptosis	[292,294]	Aging models, neuroprotection, cancer cell studies
**Sulforaphane**	Potent Nrf2 inducer → ↑ phase II enzymes, ↑ GSH synthesis, cytoprotective hormetic action	Overexposure may cause cellular stress beyond adaptive capacity	[293,301]	Neuroprotection, metabolic health, preclinical aging studies
**Quercetin**	Modulates Nrf2 and NF-κB; antioxidant at low doses, supports redox homeostasis	At high concentrations can be pro-oxidant, cytotoxic in some systems	[292,299]	Inflammatory models, neuroprotection, cancer cell lines

“Low-dose effect” and “High-dose effect” entries summarize commonly reported, dose-dependent actions observed across in vitro and in vivo preclinical studies; exact responses depend on cell type, exposure protocol, bioavailability and metabolic context. Disease models column indicates contexts in which hormetic vs. toxic effects have been studied; for cancer, high-dose pro-oxidant activity of polyphenols is sometimes exploited therapeutically to induce ferroptosis in malignant cells. Abbreviations: Nrf2, nuclear factor erythroid 2-related factor 2; GSH, glutathione; GPX4, glutathione peroxidase 4; NF-κB, nuclear factor kappa-light-chain-enhancer of activated B cells; ROS, reactive oxygen species; RCD, regulated cell death; SIRT1, sirtuin 1.

## Data Availability

No new data were created or analyzed in this study. Data sharing is not applicable to this article.

## Abbreviations

The following abbreviations are used in this manuscript:

^•^OH	hydroxyl radicals
4-HNE	4-hydroxynonenal
AA	arachidonic acid
ACSL3	acyl-CoA synthetase long-chain family member 3
ACSL4	acyl-CoA synthetase long chain family member 4
AD	Alzheimer’s disease
AdA	adrenic acid
AIFM2	apoptosis-inducing factor, mitochondrion-associated, 2
ALOX5	arachidonate 5-lipoxygenase
ALS	amyotrophic lateral sclerosis
AMPK	AMP-activated protein kinase
AOPP	advanced oxidation protein products
ARE	antioxidant response element
ATP	adenosine triphosphate
ATP7B	ATPase copper transporting beta
BAK	BCL-2-antagonist killer
BAX	BCL-2-associated X
BBC3	BCL2-binding component 3
BCL2	B-cell lymphoma 2
BCS	bathocuproine disulfonate
BID	BH3-interacting domain death agonist
BSO	buthionine sulfoximine
cGAS–STING	cyclic GMP–AMP synthase–stimulator of interferon genes
CLP	cecal ligation and puncture
CNV	copy number variation
CoQ	Coenzyme Q10
COVID-19	coronavirus disease 2019
CP	ceruloplasmin
CRG	copper death-related genes
CRGs	cuproptosis-related genes
CTR1	copper transporter 1
CVD	cardiovascular diseases
DAMPs	damage-associated molecular patterns
DEP	deferiprone
DFO	deferoxamine
DFX	deferasirox
DHODH	dihydroorotate dehydrogenase (quinone)
DLAT	dihydrolipoyl acetyltransferase
DMT1	divalent metal transporter 1
ECM	extracellular matrix
EGCG	epigallocatechin-3-gallate
ETC	electron transport chain
FDX1	ferredoxin 1
FEACR	ferroptosis-associated circRNA
Fer-1	ferrostatin-1
Fe-S	iron-sulfur
FINs	ferroptosis inducers
FOXO1	forkhead box O1
FPN1	ferroportin
FRAP	ferric reducing antioxidant power
FSP1	Ferroptosis Suppressor Protein 1
FTH1	ferritin heavy chain 1
GCSH	glycine cleavage system H-protein
GPX4	glutathione peroxidase 4
GSH	glutathione
H_2_O_2_	hydrogen peroxide
HD	Huntington’s disease
HFpEF	heart failure with preserved ejection fraction
HIF-1α	hypoxia-inducible factor 1-alpha
HMGB1	high mobility group box 1
HT22	HT-22 mouse hippocampal neuronal cell line
I/R	ischemia–reperfusion
IL-1β	interleukin-1 beta
JAK-STAT	Janus kinase–signal transducer and activator of transcription
Keap1	kelch-like ECH-associated protein 1
KGD	α-ketoglutarate dehydrogenase
LAD	left anterior descending
LC–MS	liquid chromatography-mass spectrometry
LIAS	lipoic acid synthetase
LIP	labile iron pool
LIPT1	lipoyltransferase 1
L-NAME	N(G)-Nitro-L-arginine methyl ester
L-OH	lipid alcohols
L-OOH	lipid hydroperoxides
LOXs	lipoxygenases
LPCAT3	acyl-CoA synthetase long-chain family member 4 (ACSL4)–activated, lysophosphatidylcholine acyltransferase 3
LPI	labile plasma iron
LPS	lipopolysaccharide
MCC950	N-[[(1, 7-hexahydro-s-indacen-4-yl)amino]carbonyl]-4-(1-hydroxy-1-methylethyl)-2-furansulfonamide sodium salt
MDA	malondialdehyde
mHTT	mutant huntingtin
MI	myocardial infarction
MnSOD	manganese superoxide dismutase
MT	metallothionein
mtDNA	mitochondrial DNA
mTOR	mammalian target of rapamycin
MUFAs	monounsaturated fatty acids
NAC	N-acetylcysteine
NAD	nicotinamide adenine dinucleotide
NAMPT	nicotinamide phosphoribosyltransferase
NCOA4	nuclear receptor coactivator 4
NFS1	cysteine desulfurase
NF-κB	nuclear factor kappa-light-chain-enhancer of activated B cells
NLRP3	NOD-, LRR- and pyrin domain-containing protein 3
NRF2	nuclear factor erythroid 2-related factor 2
NTBI	non-transferrin-bound iron
O2^•−^	superoxide anion
OA	osteoarthritis
OXPHOS	oxidative phosphorylation
p16INK4a (CDKN2A)	cyclin-dependent kinase inhibitor 2A
p21CIP1/WAF1 (CDKN1A)	cyclin-dependent kinase inhibitor 1A
p62	sequestosome 1
PAMPs	pathogen-associated molecular patterns
PD	Parkinson’s disease
PDC	pyruvate dehydrogenase complex
PGC-1α	peroxisome proliferator-activated receptor-gamma coactivator 1-alpha
PINK1	PTEN-induced kinase 1
PPARγ	peroxisome proliferator-activated receptor gamma
PRRs	pattern recognition receptors
Prx6	mitochondrial peroxiredoxin 6
PUFAs	polyunsaturated fatty acids
PUMA	p53 upregulated modulator of apoptosis
RA	Rheumatoid Arthritis
RAGE	receptor for advanced glycation end-products
RAS	rat sarcoma virus
RB	retinoblastoma
RCD	regulated cell death
ROS	reactive oxygen species
RSL3	(1S,3R)-methyl 2-(2-chloroacetyl)-1-(4-(methoxycarbonyl)phenyl)-2,3,4,9-tetrahydro-1H-pyrido[4-b]indole-3-carboxylate
RTAs	radical-trapping antioxidants
RVG29	rabies virus glycoprotein 29
SARS-CoV-2	severe acute respiratory syndrome coronavirus 2
SASP	senescence-associated secretory phenotype
SCD1	stearoyl-CoA desaturase-1
SEC24B	protein transport protein Sec24 homolog B, COPII coat complex component
SIRT1	sirtuin 1
SLC31A1	solute carrier family 31 (copper transporter), member 1
SLC7A11	solute carrier family 7 member 11
SNpc	substantia nigra pars compacta
TAX1BP1	Tax1-binding protein 1
TBARS	thiobarbituric acid reactive substances
TCA	tricarboxylic acid cycle
TfR1	transferrin receptor-1
TLRs	Toll-like receptors
TNF-α	tumour necrosis factor-α
TPP	thiamin pyrophosphate
TTM	tetrathiomolybdate ammonium
UCP2	uncoupling protein-2
WD	Wilson’s disease
